# OTUB2/ALYREF axis modulates the docetaxel resistance of castration-resistant prostate cancer via upregulating ABCG4-mediated drug efflux

**DOI:** 10.7150/ijbs.126319

**Published:** 2026-03-17

**Authors:** Zhi-Bin Ke, Jia-Yin Chen, Bin Lin, Chao-Ran Chen, Yu-Ting Xue, Jiang-Bo Sun, Zi-Heng Yan, Yu-Xuan Zhao, Meng-Xin Liu, Zhen Wang, Xue-Yi Xue, Qing-Shui Zheng, Yong Wei, Ning Xu

**Affiliations:** 1 Department of Urology, Urology Research Institute, the First Affiliated Hospital, Fujian Medical University, Fuzhou 350005, China.; 2 Department of Urology, National Regional Medical Center, Binhai Campus of the First Affiliated Hospital, Fujian Medical University, Fuzhou 350212, China.; 3 Fujian Key Laboratory of Tumor Immunotherapy, the First Affiliated Hospital, Fujian Medical University, Fuzhou 350005, China.

**Keywords:** castration-resistant prostate cancer, docetaxel resistance, de-ubiquitination, m5C modification, cancer-associated fibroblasts

## Abstract

Docetaxel (DTX) is a standard chemotherapy agent for castration-resistant prostate cancer (CRPC); however, DTX resistance remains a major clinical challenge, and the underlying molecular mechanisms are not fully understood. In our study, it was found that OTUB2 was highly expressed in DTX-resistant CRPC and could be served as a key driver of DTX resistance. Mechanistically, OTUB2 stabilizes the m5C reader ALYREF by removing its K48-linked polyubiquitin chains, leading to increased ALYREF protein levels. And then, ALYREF enhances the mRNA stability and expression of ABCG4, thereby promoting ATP-dependent efflux of DTX. Moreover, the expression of OTUB2 mRNA and protein could be regulated by FOXD3-AS1 derived from cancer-associated fibroblasts (CAFs). More importantly, treatment with OTUB2 inhibitor (OTUB2-IN-1) resensitized resistant CRPC to DTX. Together, our findings establish OTUB2 as a novel driver of DTX resistance in CRPC and highlight the role of CAFs-derived FOXD3-AS1 and OTUB2/ALYREF/ABCG4 axis in modulating DTX resistance of CRPC.

## Introduction

Prostate cancer (PCa) represents a significant global health burden, ranking as the most frequently diagnosed malignancy in men and the fifth leading cause of cancer-related mortality worldwide^1^. Despite established therapeutic strategies for PCa, including radical prostatectomy and radical radiotherapy, curative outcomes remain suboptimal^2^. A majority of patients ultimately progress to castration-resistant prostate cancer (CRPC)^3^, ^4^. Docetaxel (DTX)-based chemotherapy represents a first-line treatment for CRPC; however, acquired resistance is developed in most patients, ultimately leading to treatment failure and poor prognosis^5^, ^6^. The mechanisms underlying DTX resistance are multifactorial and complex. Current research has predominantly focused on aberrant activation of androgen receptor (AR) signaling^7^, ^8^, tumor microenvironment remodeling^9^, cancer stem cells plasticity^10^, ^11^, and enhanced DNA damage repair capacity^12^. Nevertheless, effective strategies to restore DTX sensitivity remain limited. Therefore, elucidating the mechanisms underlying DTX resistance in CRPC is of paramount clinical significance.

Ubiquitination, a critical post-translational modification, regulates protein stability, activity, and subcellular localization, playing indispensable roles in fundamental biological processes including DNA damage repair, proliferation, autophagy, cell cycle progression, and apoptosis^13^, ^14^. The human genome encodes approximately 100 deubiquitinating enzymes (DUBs). Among these, the ovarian tumor (OTU) family represents a major DUB class that recognizes and hydrolyzes diverse ubiquitin chain linkages^15^. Emerging evidence has implicated OTUB2 in tumor progression across various malignancies. Specifically, OTUB2 silencing destabilized sorting nexin 29 pseudogene 2 (SNX29P2) and promoted ovarian cancer development^16^. In gastric cancer, OTUB2 enhanced KRT80 stability via de-ubiquitination, thereby facilitating tumor proliferation^17^. Moreover, pharmacological inhibition of OTUB2, significantly reduced the expression of PD-L1 in cancer cells and suppressed cancer growth by interfering with its de-ubiquitinase activity^18^. However, the functional role and molecular mechanisms of OTUB2 in CRPC DTX resistance remain unexplored.

Cancer-associated fibroblasts (CAFs) have recently emerged as critical regulators of chemotherapy resistance in multiple malignancies, including pancreatic cancer^19^, rectal cancer^20^, breast cancer^21^, esophageal cancer^22^, gastric cancer^23^. Regarding DTX resistance in PCa, Xiong, *et al.*^24^. demonstrated that CAFs regulated the DTX chemoresistance via modulating ANGPTL4-IQGAP1 axis of PCa. Similarly, Zhao, *et al.*^25^. found that CAFs inhibited ferroptosis and facilitated DTX resistance of PCa via regulating miR-432-5p/CHAC1. However, the involvement of CAFs-derived long non-coding RNAs (lncRNAs) in the DTX resistance of PCa remains poorly characterized.

In this study, we discovered that OTUB2 conferred the docetaxel resistance of CRPC via removing the K48-linkage poly-ubiquitination of ALYREF, thereby stabilizing ALYREF protein both *in vitro* and *in vivo*. The m5C reader ALYREF subsequently upregulated ABCG4 mRNA stability, facilitating ATP-dependent DTX efflux. Furthermore, we identify a novel regulatory mechanism wherein exosomal FOXD3-AS1, derived from CAFs, is transferred to CRPC cells and upregulates the mRNA and protein expression of OTUB2 through miR-5586-5p sponging, consequently promoting DTX resistance. Collectively, our results revealed a novel driver for docetaxel resistance of CRPC and highlighted the pivotal role of OTUB2 and CAFs in modulating DTX resistance of CRPC.

## Materials and Methods

### Cell culture

Human CRPC cell lines (22RV1 and DU145) and HEK-293T were authenticated by STR DNA profiling and tested negative for mycoplasma contamination by PCR. All cells were maintained in a humidified atmosphere at 37 °C with 5% CO_2_. Culture media were supplemented with 10% fetal bovine serum (FBS) and 1% Penicillin-Streptomycin-Gentamicin Solution (Solarbio, Beijing, China). DU145 cells in MEM medium, 22RV1 cells in RPMI-1640 medium.

### Establishment of DTX-resistant CRPC cell lines

We generated DTX-resistant CRPC cell lines (22RV1R and DU145R) using an intermittent, stepwise dose-escalation protocol as previously described^26^, ^27^. Upon reaching 85-95% confluence, parental DU145 and 22RV1 cells were exposed to 0.1 nM DTX for 24 h, followed by recovery in drug-free medium until normal growth resumed. The DTX concentration was progressively increased in a stepwise manner: 0.2, 0.5, 0.75, 1.0, 1.5, 2.0, 5.0, 7.5, 10.0, 15.0, 20.0, 40.0 nM. Following six months of continuous exposure, the resulting resistant sublines were designated as 22RV1R and DU145R. To confirm their resistance, we measured the DTX IC50 value and colony-forming capacity under DTX treatment.

### RNA extraction and RT-qPCR analysis

Total RNA was extracted from cells using the Total RNA Isolation Kit V2 (Vazyme Biotech, Nanjing, China) according to the manufacturer's instructions. RNA concentration and purity were assessed by a NanoDrop spectrophotometer. Complementary DNA (cDNA) was synthesized from 1 µg of total RNA using the HiScript III RT SuperMix for quantitative real-time PCR (RT-qPCR) (Vazyme Biotech, Nanjing, China). RT-qPCR was performed with SYBR Green Master Mix on a QuantStudio 5 Real-Time PCR System (Applied Biosystems, USA). Gene expression levels were normalized to GAPDH as an internal control using the 2^-ΔΔCt^ method.

### Plasmids transfection, RNA inference, and lentivirus transduction

Plasmid or lentiviruses carrying sgRNA or shRNA targeting FOXD3-AS1, or OTUB2, or ALYREF were obtained from Shanghai GENECHEM Co., Ltd. (Shanghai, China). For plasmid transfection, cells were seeded to 50%-60% confluence and transfected using Lipofectamine 2000 (Thermo Fisher Scientific) according to the manufacturer's protocol. Lentiviral transduction was performed using Polybrene following the manufacturer's instructions. Stable cell lines were selected using puromycin (2 μg/mL) for 2 weeks.

### Transcriptome sequencing

Total RNA was extracted from both the treated and control groups. RNA quality and integrity were assessed using a NanoDrop spectrophotometer and Agilent Bioanalyzer. Samples were sent for high-throughput RNA sequencing with a sequencing depth of 60M reads (6G). To ensure the comparability between replicates, batch effect correction was applied to normalized count matrices using the limma R package.

### RNA immunoprecipitation sequencing (RIP-seq) and RIP-qPCR

Cells were lysed in RIP lysis buffer supplemented with protease inhibitors, RNase inhibitor, and PMSF. Ten percent of the lysate was reserved as input control, and the remaining lysate was centrifuged to collect the supernatant. Pre-cleared supernatant was incubated with Protein A/G magnetic beads pre-bound with primary antibody or control IgG overnight at 4 ℃. Beads were washed five times with RIP wash buffer, and bound RNA was eluted at 65 ℃ for 15 min, treated with Proteinase K (55 ℃, 30 min), and extracted using TRIzol. The purified RNA was subjected to either high-throughput sequencing (RIP-seq) or reverse transcription followed by RT-qPCR (RIP-qPCR). RIP-seq libraries were sequenced at approximately 40 million reads per sample.

### m5C-MeRIP-qPCR

Cells were lysed in m5C-MeRIP lysis buffer, with approximately 10% of total RNA reserved as input control. The remaining lysate was centrifuged, and total RNA was fragmented at 70 ℃ for 15 min to generate 100-300 nt fragments, followed by purification. Protein A beads were incubated with anti-m5C antibody or control IgG at 4 ℃ for 2 h, and then mixed with fragmented RNA overnight at 4 ℃ with gentle rotation. After five washes with m5C-MeRIP buffer, bound RNA was extracted using TRIzol reagent. cDNA synthesis and RT-qPCR were performed as described using primers targeting the putative m5C sites in ABCG4 transcript.

### mRNA stability assay

To assess ABCG4 mRNA stability, cells in the logarithmic growth phase were treated with the transcription inhibitor actinomycin D (0.5 μg/mL) and harvested at the indicated time points (0, 1, 2, 3, and 4 h). Total RNA was extracted, and the relative abundance of ABCG4 mRNA was measured by RT-qPCR. The remaining mRNA of ABCG4 and a stable internal reference gene at each time point were quantified and expressed as a percentage of the level at 0 h (set to 100%).

### Western blot analysis

Proteins were extracted using RIPA lysis buffer supplemented with protease inhibitor, phosphatase inhibitor, and PMSF. Protein concentrations were determined using a BCA assay kit. Protein was separated by SDS-PAGE and transferred onto PVDF membranes. The membranes were then blocked with NcmBlot blocking buffer for one hours, followed by overnight incubation at 4℃ with primary antibodies. After washing with Tris-buffered saline containing 0.1% Tween-20 (TBST), membranes were incubated with HRP-conjugated secondary antibodies for 1 h at room temperature. We then used an ECL reagent for visualizing protein bands. Primary antibodies used were: anti-OTUB2 (1:1000; proteintech; 12066-1-AP), anti-ALYREF (1:1000; abcam; ab202894), anti-ABCG4 (1:1000; proteintech; 14269-1-AP), anti-tubulin (1:2000; proteintech; 11224-1-AP), anti-β-actin (1:2000; proteintech; 20536-1-AP), anti-GAPDH (1:2000; proteintech; 60004-1-Ig), anti-polyubiquitin (1:1000; CST; 58395), anti-K48 linkage specific polyubiquitin (1:1000; CST; 8081S), anti-HA (1:1000; proteintech; 51064-2-AP), anti-Flag (1:1000; sigma; F1804), anti-His (1:1000; proteintech; 10001-0-AP).

### Co-immunoprecipitation (Co-IP) assay

Cells were lysed to extract total proteins. To reduce nonspecific binding, the cell lysates were pre-cleared by incubating with Protein A/G magnetic beads for 1 h at 4°C. Beads were then removed by centrifugation, and the cleared lysates were incubated overnight at 4°C with 2-5 µg of specific antibody or an appropriate isotype control antibody. Protein A/G magnetic beads were added to the immunocomplexes and incubated for an additional 2 h at 4°C. The immunoprecipitated complexes were washed 4 times with lysis buffer, and then heated with loading buffer at 95°C for 5 minutes, and then analyzed by western blotting.

### De-ubiquitination assay

Cells were treated with 10 µM MG132 (a proteasome inhibitor) for 6 h to prevent protein degradation. Total proteins were extracted using RIPA buffer containing protease inhibitors. Immunoprecipitation was performed using specific primary antibodies. The ubiquitination levels of ALYREF were then assessed by western blotting.

### Dual-luciferase reporter assay

The 3'-UTR sequence or specific fragments of the targeted genes were cloned into the pGL3 luciferase vector by Shanghai GenePharma Co., Ltd. These plasmids were co-transfected into HEK-293T cells along with either miR-5586-5p mimics or miR-5586-5p inhibitor using Lipofectamine 2000 reagent (Thermo Fisher Scientific). After 48 h of transfection, cells were harvested for luciferase activity measurement using the Dual-Luciferase Reporter Assay Kit (Beyotime, China) according to the manufacturer's protocol. Firefly luciferase activity was normalized to Renilla luciferase activity.

### Immunofluorescence

Cells were seeded onto coverslips in six-well plates 24 h prior to treatment. Following treatment, cells were fixed with 4% paraformaldehyde (PFA) for 30 min, and then permeabilized with 0.1% Triton X-100 for 30 min. To block nonspecific binding, cells were incubated with 2% goat serum for 30 min at room temperature. Cells were then incubated with primary antibodies overnight at 4°C. Next, secondary antibodies were applied and incubated for 1h at room temperature. Following additional PBS washes, cells were stained with DAPI for approximately 2 min at room temperature. Finally, cells were mounted with anti-fade solution, and fluorescence images were captured using an Olympus fluorescence microscope.

### Isolation of primary fibroblasts and purification of exosomes

Fibroblasts were isolated using a previously described method^24^. Briefly, we minced and dissociated the tumor tissues in 1640 medium containing 10% FBS and 0.5% collagenase Type I at 37 °C for 2 h. Isolated fibroblasts were cultured in 1640 medium containing 10% FBS at 37°C with 5% CO₂ until adherent. We identified CAFs according these criteria: spindle morphology, flow cytometry confirming the EPCAM⁻, CD45⁻, CD31⁻, PDGFRα⁺, western blot confirming increased expression of a-SMA, FAP, Vimentin, PDGFRa. Only low passage (<5 passages) fibroblasts were utilized for our experiments. Exosomes were isolated from the culture supernatants of CAFs or normal fibroblast (NFs) by differential centrifugation. The medium was first centrifuged at low speed to remove cells and debris, followed by high-speed centrifugation to collect the exosomes. Exosome morphology was examined by transmission electron microscopy. Size distribution was measured using the NanoSight NS300 system. Exosome identity was confirmed by western blotting for the markers CD63 and TSG101.

### Colony formation assay

Exponentially growing cells were seeded into 6-well plates at a density of 5×10^2^ cells per well and cultured for 14 days in the presence of DTX. The colonies were fixed and stained with 0.5% crystal violet for 15 minutes at room temperature. Colonies containing more than 50 cells were counted and photographed.

### CCK-8 toxicity assay

For CCK-8 toxicity assay, cells were seeded into 96-well plates at a density of 5×10^3^ cells per well, with five replicates per group. After 24 hours, cells were incubated with varying concentrations of DTX (2.5nM, 5nM, 10nM, 15nM, 20nM, 25nM, 30nM, 35nM, 40nM) for 24 h. Subsequently, 100 μL CCK-8 solution (10 μL CCK-8 regent and 90 μL complete medium) was added to each well. The plates were incubated at 37°C for 1 h, and the optical density (OD) at 450 nm was measured using a microplate reader.

### Cell line-derived xenograft (CDX) and patient-derived xenograft (PDX) models

We purchased 4-6 weeks-old male athymic BALB/c nude mice from Shanghai SLAC Laboratory Animal Co., Ltd. All mice were castrated surgically. For the orthotopic CDX model, 1×10⁶ CRPC cells suspended in PBS containing Matrigel were injected directly into the mouse prostate using a 26-gauge needle under anesthesia. CDX tumor-bearing mice were administered DTX via intraperitoneal injection every 3 days, starting on day 12 post-injection of the tumor cells. When we verify the role of CAF exosomal FOXD3-AS1 *in vivo*, CAF and tumor cells were fully mixed with the proportion of 1:1 before injection into mice^24^. For subcutaneous PDX model, CRPC PDXs were transplanted subcutaneously into flank of mice. The PDX tumors were observed and measured every 3 days. PDX tumor-bearing mice were administered DTX via intraperitoneal injection every 3 days, starting when tumors reached 50-60 mm^3^. Tumor volume was calculated using the following formula: π/6×a×b^2^ (a: length; b: width, a > b).

### Establishment of primary CRPC cell lines

Fresh CRPC PDX tissues were obtained and cut into small pieces (approximately 1 mm^3^). Then, we digested CRPC PDX pieces to obtain single-cell suspension. Only low passage (<5 passages) of primary cells were utilized for our experiments.

### Patient-derived organoid (PDO) models

Patient-derived CRPC tissue specimens were enzymatically digested and cultured in Matrigel-based 3D matrices. After reaching nearly 100 μm of diameter, organoids in all groups were treated with DTX of indicated concentration. Size and morphology were monitored microscopically. After 48 h, the cell viability of organoids was detected. We transfected the C51S OTUB2 plasmid to CRPC organoids with OTUB2 knockdown using shRNA to construct OTUB2 C51S organoids.

### Clinical specimens

We obtained fresh CRPC tissues by prostate biopsy and fresh HSPC tissues by radical prostatectomy from our center for paraffin embedded wax blocks, or western blot, or RT-qPCR, or organoids culture, or constructing PDX model. All patients provided written informed consent before sample collection.

### Statistical analysis

The statistical analyses for data were conducted by GraphPad Prism 9.0 (GraphPad Prism Software, Inc., USA) and SPSS version 26.0 software (IBM, Corp, Armonk, NY, USA). Quantitative data following a normal distribution were indicated as mean ± standard deviation (SD), and comparisons between groups were analyzed using one-way ANOVA or Students t-test. Repeated measures ANOVA was performed to assess repeated measurement data. Survival analysis was conducted by Kaplan-Meier methods. *P* < 0.05 was regarded as statistically significant difference.

## Results

### OTUB2 is highly expressed in DTX-resistant CRPC

**Figure 1:**
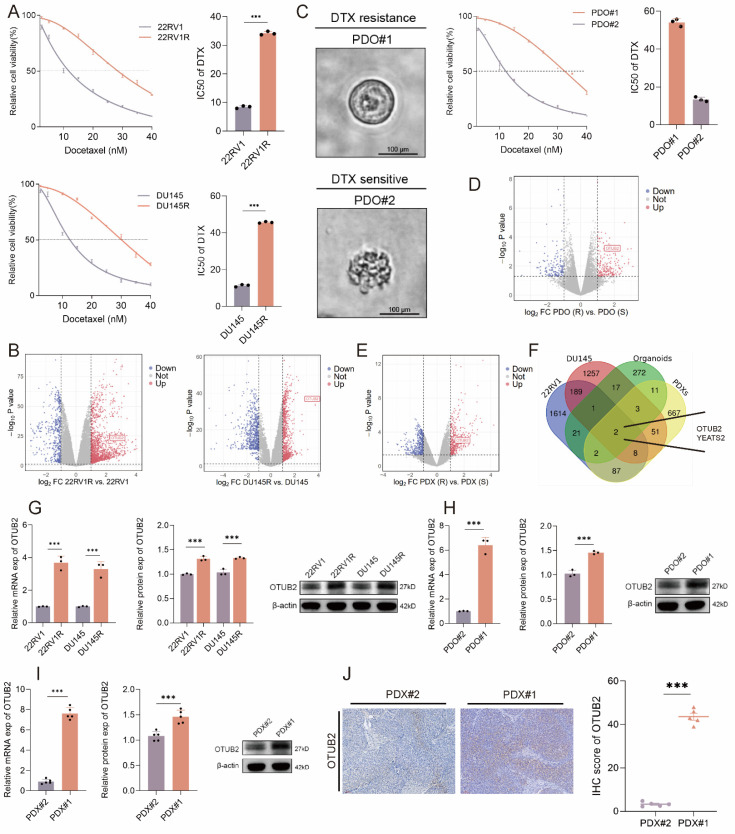
** OTUB2 was highly expressed in DTX-resistant CRPC.** (**A**)The IC50 value of DTX showing the successful construction of DTX-resistant (22RV1R, DU145R) CRPC cells; three biological replicates were conducted; unpaired two-tailed student's t-test. (**B**) Volcano map showing the differentially expressed genes (DEGs) by comparing the RNA-seq results between DTX-resistant and DTX-sensitive CRPC cells (DU145R vs. DU145; 22RV1R vs. 22RV1). (**C**) Representative bright-field images of organoids from two cases of CRPC patients. The CRPC organoids were treated with DTX for 48 hours in a gradient concentration and then the IC50 value of DTX was evaluated; three biological replicates were conducted; unpaired two-tailed student's t-test. (**D**) Volcano map showing the DEGs by comparing the RNA-seq results between DTX-resistant and DTX-sensitive CRPC organoids. (**E**) Volcano map showing the DEGs by comparing the RNA-seq results between DTX-resistant and DTX-sensitive CRPC PDXs. (**F**) Venn diagram showing the intersection genes of the four above libraries. (**G**) RT-qPCR and western blot indicating the differential expression of OTUB2 mRNA and protein between DTX-resistant and DTX-sensitive CRPC cells; three biological replicates were conducted; unpaired two-tailed student's t-test. (**H**) RT-qPCR and western blot indicating the differential expression of OTUB2 mRNA and protein between DTX-resistant and DTX-sensitive CRPC organoids; three biological replicates were conducted; unpaired two-tailed student's t-test. (**I**) RT-qPCR and western blot indicating the differential expression of OTUB2 mRNA and protein between DTX-resistant and DTX-sensitive CRPC PDXs; three biological replicates were conducted; unpaired two-tailed student's t-test. (**J**) IHC staining showing the differential expression level of OTUB2 protein between DTX-resistant and DTX-sensitive CRPC PDXs; unpaired two-tailed student's t-test. *P<0.05, **P<0.01, ***P<0.001.

DTX resistance remains a significant challenge for CRPC treatment. To identify critical drivers of this resistance, we established the DTX-resistant CRPC cell lines (named 22RV1R and DU145R, respectively) through stepwise dose escalation. Successful establishment was confirmed by increased IC50 values and enhanced colony formation under DTX treatment (**Figure 1A; Figure S1A-B**). RNA-seq between DTX-resistant and parental CRPC cells was conducted (DU145R vs. DU145; 22RV1R vs. 22RV1) to investigate the drivers for DTX resistance (**Figure 1B**). Next, we generated two cases of CRPC organoids using tissues from prostate biopsy. Based on their IC50 value, we classified these organoids as DTX-resistant (PDO#1) or DTX-sensitive (PDO#2) (**Figure 1C**). RNA-seq between DTX-resistant and DTX-sensitive CRPC organoids was also conducted (**Figure 1D**). Besides, we successfully constructed the two cases of CRPC PDX (PDX#1 and PDX#2) using tissues from prostate biopsy. These two CRPC PDXs were subcutaneously transplanted into the flank of male BALB/c nude mice. When DTX was injected intraperitoneally every 3 days, the tumor growth of PDX#2 was significantly slower than that of PDX#1, and the tumor weights and volumes of PDX#2 were smaller than that of PDX#1 (**Figure S1C-E**). Hence, we classified PDX#1 as the DTX-resistant PDXs and PDX#2 as the DTX-sensitive PDXs, respectively. RNA-seq between DTX-resistant and DTX-sensitive CRPC PDXs was conducted to investigate the drivers for the DTX resistance of CRPC (**Figure 1E**). The overlap genes after the intersection of the four above libraries were presented in **Figure 1F**, which showed that OTUB2 and YEATS2 might be vital drivers for the DTX resistance of CRPC. For the reason that the fold change of OTUB2 is higher than YEATS2 in the above all four datasets, and that the knockdown of YEATS2 could not significantly affect the IC50 of 22RV1R and DU145R (**Figure S1F-G**), we selected OTUB2 for further experiments. The results of RT-qPCR and western blot indicated that the expression level of OTUB2 mRNA and protein in DTX-resistant CRPC cells was significantly higher than that in DTX-sensitive CRPC cells (**Figure 1G**). The results of RT-qPCR and western blot indicated that the expression level of OTUB2 mRNA and protein in DTX-resistant CRPC organoids was significantly higher than that in DTX-sensitive CRPC organoids (**Figure 1H**). The results of RT-qPCR, western blot, and IHC staining indicated that the expression level of OTUB2 mRNA and protein in DTX-resistant CRPC PDXs was significantly higher than that in DTX-sensitive CRPC PDXs (**Figure 1I-J**). These results demonstrated that the mRNA and protein of OTUB2 was highly expressed in DTX-resistant CRPC compared with that in DTX-sensitive CRPC and OTUB2 might be a vital driver for the DTX resistance of CRPC.

### Tumoral OTUB2 confers the DTX resistance of CRPC

**Figure 2:**
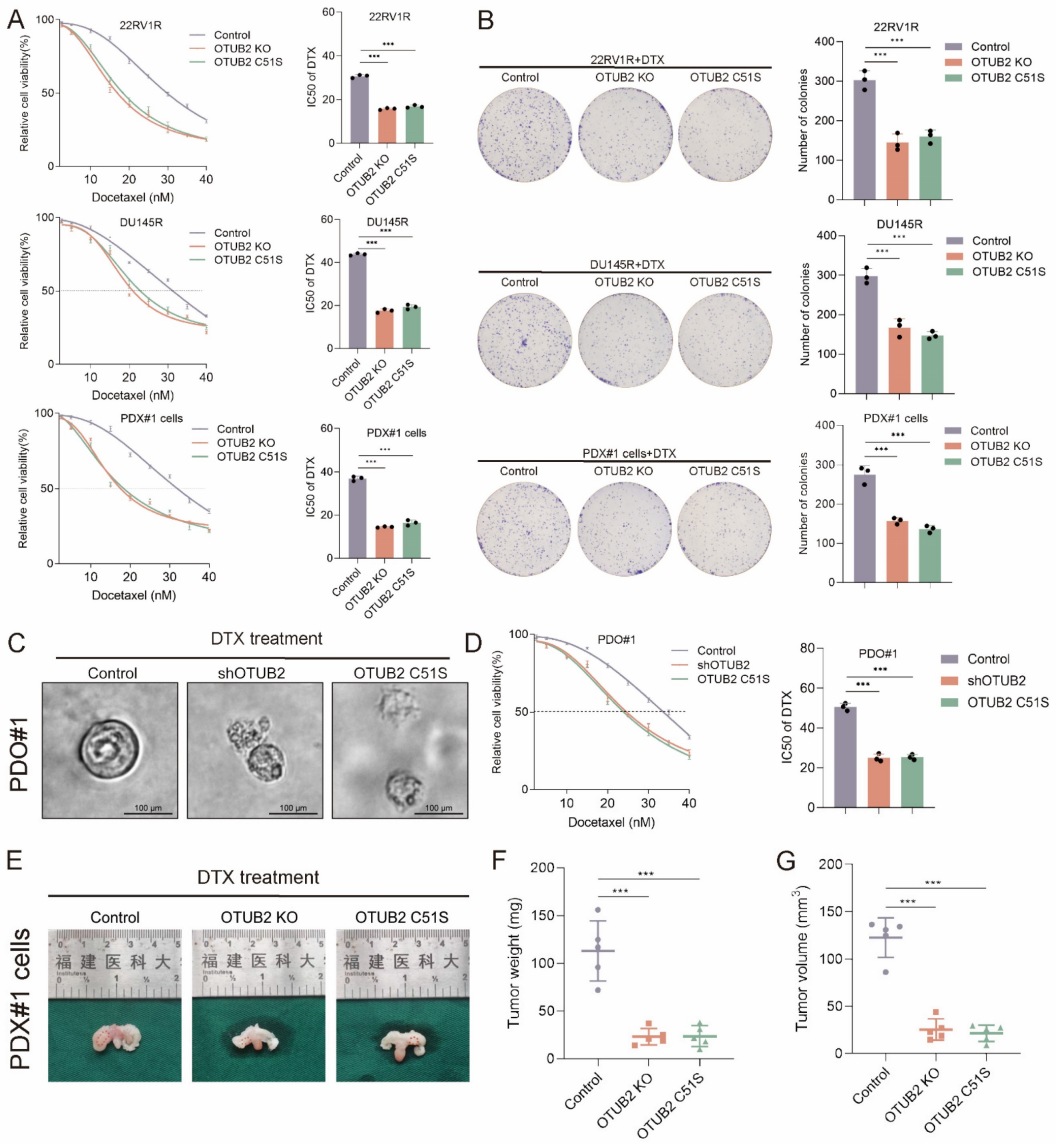
** Tumoral OTUB2 confers the DTX resistance of CRPC *in vitro* and *in vivo*.** The effect of OTUB2 knockout or dead mutation (C51S) on the IC50 value of DTX of 22RV1R, DU145R, and PDX#1 derived primary cells (PDX#1 cells); three biological replicates were conducted; one-way ANOVA test. (**B**) Representative images and quantitative results of cloning formation assay showing the effect of OTUB2 knockout or dead mutation (C51S) on the cloning formation ability of 22RV1R, DU145R, and PDX#1 cells; three biological replicates were conducted; one-way ANOVA test. (**C**) Representative bright-field images of CRPC organoids showing the effect of OTUB2 knockdown or dead mutation (C51S) on the morphological characteristics of DTX-resistant organoids (PDO#1) under DTX treatment. (**D**) The effect of OTUB2 knockdown or dead mutation (C51S) on the IC50 value of DTX of DTX-resistant organoids (PDO#1); three biological replicates were conducted; one-way ANOVA test. (**E-G**) Stable PDX#1 cells of indicated groups were injected into the orthotopic prostate of male BALB/c nude mice. DTX was injected intraperitoneally every 3 days from day 12 onwards. Tumor images, weight and volume were obtained after dissection; one-way ANOVA test. *P<0.05, **P<0.01, ***P<0.001.

To investigate the role of OTUB2 in DTX resistance of CRPC, we isolated DTX-resistant primary CRPC cells from PDX xenograft (named PDX#1 cells). We constructed the stable 22RV1R, DU145R, PDX#1 cells of OTUB2 knockout or dead mutation (C51S). The results of CCK8 toxicity assay showed that the knockout or dead mutation (C51S) of OTUB2 significantly decreased the IC50 value of DTX of 22RV1R, DU145R, PDX#1 cells (**Figure 2A**). The results of cloning formation assay showed that the knockout or dead mutation (C51S) of OTUB2 significantly inhibited the cloning formation ability of 22RV1R, DU145R, PDX#1 cells under DTX treatment (**Figure 2B**). Besides, the IC50 of organoids showed that the knockdown or dead mutation (C51S) of OTUB2 significantly decreased the IC50 value of DTX of DTX-resistant organoids (PDO#1) (**Figure 2C-D**). These results revealed that high expression and the de-ubiquitination activity of tumoral OTUB2 promoted the DTX resistance of CRPC *in vitro*.

Next, we explored the role of OTUB2 in DTX resistance of CRPC *in vivo*. The stable PDX#1 cells of indicated groups were injected into the orthotopic prostate of male BALB/c nude mice. DTX was injected intraperitoneally every 3 days from day 12 onwards. The tumor weights and volumes in OTUB2 knockout or dead mutation (C51S) group were smaller than that in the control group (**Figure 2E-G**). Moreover, PDX#1 were transplanted subcutaneously into the flank of male BALB/c nude mice. The lentivirus carrying OTUB2 shRNA was injected into tumors. DTX was injected intraperitoneally every 3 days, starting after the tumor reaching 50-60 mm^3^. The tumor growth of PDX#1 in shOTUB2 group was significantly slower than that in the control group, and the tumor weights and volumes in shOTUB2 group were smaller than that in the control group (**Figure S1C-E**). The stable DU145R cells of indicated groups were injected into the orthotopic prostate of male BALB/c nude mice. DTX was injected intraperitoneally every 3 days from day 12 onwards. The tumor weights and volumes in OTUB2 knockout or dead mutation (C51S) group were smaller than that in the control group (**Figure S1H-J**). These results revealed that high expression and the de-ubiquitination activity of tumoral OTUB2 promoted the DTX resistance of CRPC *in vivo*.

### OTUB2 deubiquitinates and stabilizes ALYREF

**Figure 3:**
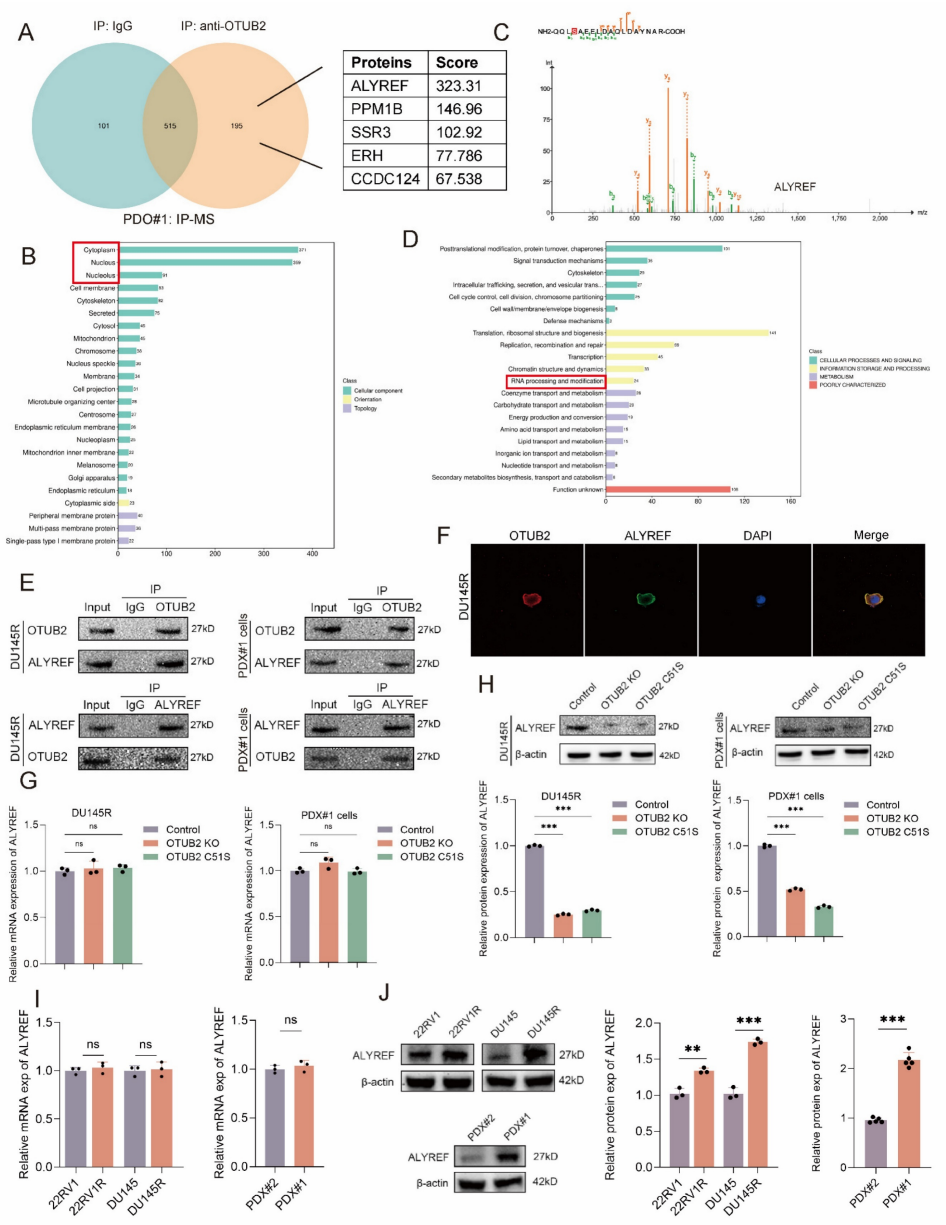
** OTUB2 bound to and regulated the expression of ALYREF protein in DTX-resistant CRPC.** (**A**) Mass spectrometry after immunoprecipitation using DTX-resistant organoids (PDO#1) identifying the binding proteins of OTUB2, and the top five proteins were ALYREF, PPM1B, SSR3, ERH, and CCDC124. (**B**) Subcellular localization of all potential binding proteins of OTUB2 identified by mass spectrometry. (**C**) Identification of ALYREF as the potential binding protein of OTUB2. (**D**) COG annotation analysis results of potential binding proteins of OTUB2 identified by mass spectrometry. (**E**) The endogenous interaction between OTUB2 and ALYREF was detected in DTX-resistant CRPC cells (DU145R and PDX#1 cells) by co-immunoprecipitation and western blotting. (**F**) Co-localization of OTUB2 and ALYREF was visualized in DTX-resistant CRPC cells (DU145R) via immunofluorescence. (**G**) RT-qPCR assay showing the effect of OTUB2 knockout or dead mutation (C51S) on the expression level of ALYREF mRNA in DTX-resistant CRPC cells (PDX#1 cells and DU145R); three biological replicates were conducted; one-way ANOVA test. (**H**) Western blot showing the effect of OTUB2 knockout or dead mutation (C51S) on the expression level of ALYREF protein in DTX-resistant CRPC cells (PDX#1 cells and DU145R); three biological replicates were conducted; one-way ANOVA test. (**I**) RT-qPCR assay showing the differential expression of ALYREF mRNA between DTX-sensitive and DTX-resistant CRPC cells and PDXs; unpaired two-tailed student's t-test. (**J**) Western blot showing the differential expression of ALYREF protein between DTX-sensitive and DTX-resistant CRPC cells and PDXs; unpaired two-tailed student's t-test. *P<0.05, **P<0.01, ***P<0.001.

To investigate the mechanism of OTUB2 promoting CRPC DTX resistance, the protein products were analyzed using mass spectrometry after immunoprecipitation of OTUB2 in DTX-resistant organoids (PDO#1). The most common subcellular localizations of potential binding proteins of OTUB2 identified by mass spectrometry were cytoplasm and nucleus. The top five proteins were ALYREF, PPM1B, SSR3, ERH, and CCDC124, of which ALYREF has a highest combining score (**Figure 3A-C; Figure S2**). The results of COG annotation analysis of the mass spectrometry showed that potential binding proteins of OTUB2 was enriched in RNA processing and modification (**Figure 3D**). Hence, we selected ALYREF as the potential binding proteins of OTUB2 in DTX-resistant CRPC. The endogenous interaction between OTUB2 and ALYREF was detected in DTX-resistant CRPC cells (PDX#1 cells and DU145R) by co-immunoprecipitation and western blotting (**Figure 3E**). The results of immunofluorescence showed that there was a co-localization of OTUB2 and ALYREF in DTX-resistant CRPC cells (DU145R) (**Figure 3F**). Furthermore, the results of RT-qPCR revealed that the knockout or dead mutation (C51S) of OTUB2 in DTX-resistant CRPC cells (PDX#1 cells and DU145R) failed to affect the expression level of ALYREF mRNA (**Figure 3G**); however, the results of western blot revealed that the knockout or dead mutation (C51S) of OTUB2 in DTX-resistant CRPC cells (PDX#1 cells and DU145R) could significantly reduce the expression level of ALYREF protein (**Figure 3H**). Moreover, the results of RT-qPCR and western blot showed that the protein rather than mRNA expression of ALYREF was highly expressed in DTX-resistant CRPC cells and PDXs compared with that in DTX-sensitive CRPC cells and PDXs (**Figure 3I-J**). These results suggested that OTUB2 specifically interacted with ALYREF protein and affected the expression level of ALYREF protein but not mRNA.

**Figure 4:**
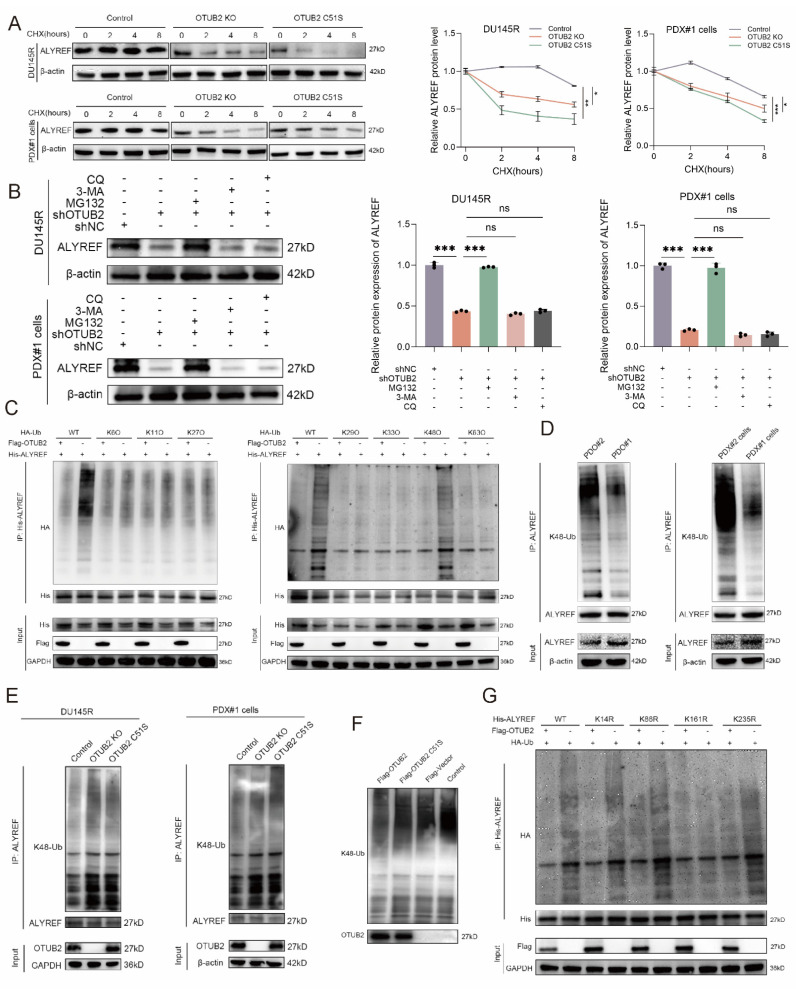
** OTUB2 regulated the K48 linkage poly-ubiquitination of ALYREF in DTX-resistant CRPC.** (**A**) Stability analysis of ALYREF protein in DTX-resistant CRPC cells (PDX#1 cells and DU145R) of indicated group, and treated with 40 μM cycloheximide (CHX); three biological replicates were conducted; two-way repeated measures ANOVA test. (**B**) Western blot analysis showing the effect of OTUB2 knockdown on the ALYREF protein levels could be rescued by MG132 but not 3-MA and CQ; three biological replicates were conducted; one-way ANOVA test. (**C**) Exogenous de-ubiquitination assay of His-ALYREF in HEK-293T cells co-transfected with His-ALYREF, Flag-OTUB2 together with HA-Ub, or HA-Ub-K6O, or HA-Ub-K11O, or HA-Ub-K27O, or HA-Ub-K29O, or HA-Ub-K33O, or HA-Ub-K48O, or HA-Ub-K63O, and treated with MG132 (10μM). (**D**) Endogenous de-ubiquitination assay showing the difference of K48 linkage poly-ubiquitination levels of ALYREF between DTX-resistant and DTX-sensitive CRPC cells or organoids (PDO#1 vs. PDO#2; PDX#1 cells vs. PDX#2 cells) treated with MG132 (10 μM). (**E**) Endogenous de-ubiquitination assay showing the effect of OTUB2 knockout or dead mutation (C51S) on the K48 linkage poly-ubiquitination of ALYREF in DTX-resistant CRPC cells (PDX#1 cells and DU145R) treated with MG132 (10 μM). (**F**)* In vitro* de-ubiquitination assay of ubiquitinated His-ALYREF protein with purified Flag-OTUB2 or Flag-OTUB2 C51S. (**G**) Exogenous ubiquitination assay of His-ALYREF in HEK-293T cells co-transfected with HA-Ub, Flag-OTUB2 together with His-ALYREF (including WT, K14R, K86R, K161R, K235R), and treated with MG132 (10μM). *P<0.05, **P<0.01, ***P<0.001.

Next, we explored whether OTUB2 could remove the conjugated ubiquitin from ALYREF protein and regulate the stability of the ALYREF protein. Notably, we found that the knockout or dead mutation (C51S) of OTUB2 led to a prominent decrease in the stability of endogenous ALYREF protein in DTX-resistant CRPC cells (PDX#1 cells and DU145R), whereas the stability of β-actin was not affected (**Figure 4A**). Besides, the results of western blot showed that the effect of OTUB2 knockdown on the protein expression of ALYREF in DTX-resistant CRPC cells (PDX#1 cells and DU145R) could be rescued by MG132 but not 3-MA or CQ, which indicated that ALYREF was degraded via ubiquitin-proteasome pathway (**Figure 4B**). There were seven lysine in ubiquitin that could form a polyubiquitin chain, including K6, K11, K27, K29, K33, K48, K63^28^. We then mutated all these above lysines on the ubiquitin to arginine except K6, or K11, or K27, or K29, or K33, or K48, or K63 (named K6O, K11O, K27O, K29O, K33O, K48O, K63O, respectively) and co-transfected His-ALYREF, Flag-OTUB2, and HA-Ub (wide or mutated type) into 293T cells. Our results revealed that it was K48 lysine residue involved in the OTUB2-medicated de-ubiquitination of ALYREF (**Figure 4C**). Endogenous *in vivo* ubiquitination assay demonstrated that the K48 linkage polyubiquitination level of ALYREF was markedly decreased in DTX-resistant CRPC compared with that in DTX-sensitive CRPC (PDX#1 cells vs. PDX#2 cells; PDO#1 vs. PDO#2) (**Figure 4D**). Furthermore, endogenous *in vivo* ubiquitination assay revealed that the K48 linkage polyubiquitination level of ALYREF was elevated significantly after OTUB2 knockout or dead mutation (C51S) in DTX-resistant CRPC cells (PDX#1 cells and DU145R) (**Figure 4E**). Next, we found that OTUB2 rather than OTUB2 C51S could also significantly promote the *in vitro* ALYREF K48 linkage de-ubiquitination (**Figure 4F**). We used UniPort database to predict the potential ubiquitinated lysine residues with gold criterion (K14, K86, K161, K235). To confirm which lysine residues of ALYREF are involved in OTUB2-mediated de-ubiquitination process of ALYREF, we mutated above lysine on the ALYREF to arginine (named K14R, K86R, K161R, K235R, respectively) and co-transfected His-ALYREF (wide or mutated type), Flag-OTUB2, and HA-Ub into 293T cells. The results showed that it was K161 lysine residue involved in the OTUB2-medicated de-ubiquitination of ALYREF (**Figure 4G**). These results suggested that OTUB2 increased the stability of the ALYREF protein by removing K48-linkage poly-ubiquitination.

### OTUB2 confers the DTX resistance of CRPC via upregulating ALYREF

We investigated whether OTUB2 promoted DTX resistance of CRPC via regulating ALYREF. The results of CCK8 toxicity assay showed that the effect of OTUB2 knockout on the IC50 value of DTX of 22RV1R, DU145R, PDX#1 cells could be rescued by the transduction of ALYREF (**Figure S3A**). The results of cloning formation assay showed that the effect of OTUB2 knockout on the cloning formation ability of 22RV1R, DU145R, PDX#1 cells under DTX treatment could be rescued by the transduction of ALYREF (**Figure S3B**). The stable DU145R cells of indicated groups were injected into the orthotopic prostate of male BALB/c nude mice. DTX was injected intraperitoneally every 3 days from day 12 onwards. The tumor weights and volumes in OTUB2 knockout group were smaller than that in the control group and could be rescued by the transduction of ALYREF (**Figure S3C-E**). These results revealed that high expression of tumoral OTUB2 promoted the DTX resistance of CRPC via upregulating ALYREF *in vitro* and *in vivo*.

### ALYREF upregulated the mRNA stability of ABCG4 in a NSUN2-mediated m5C-dependent manner

**Figure 5:**
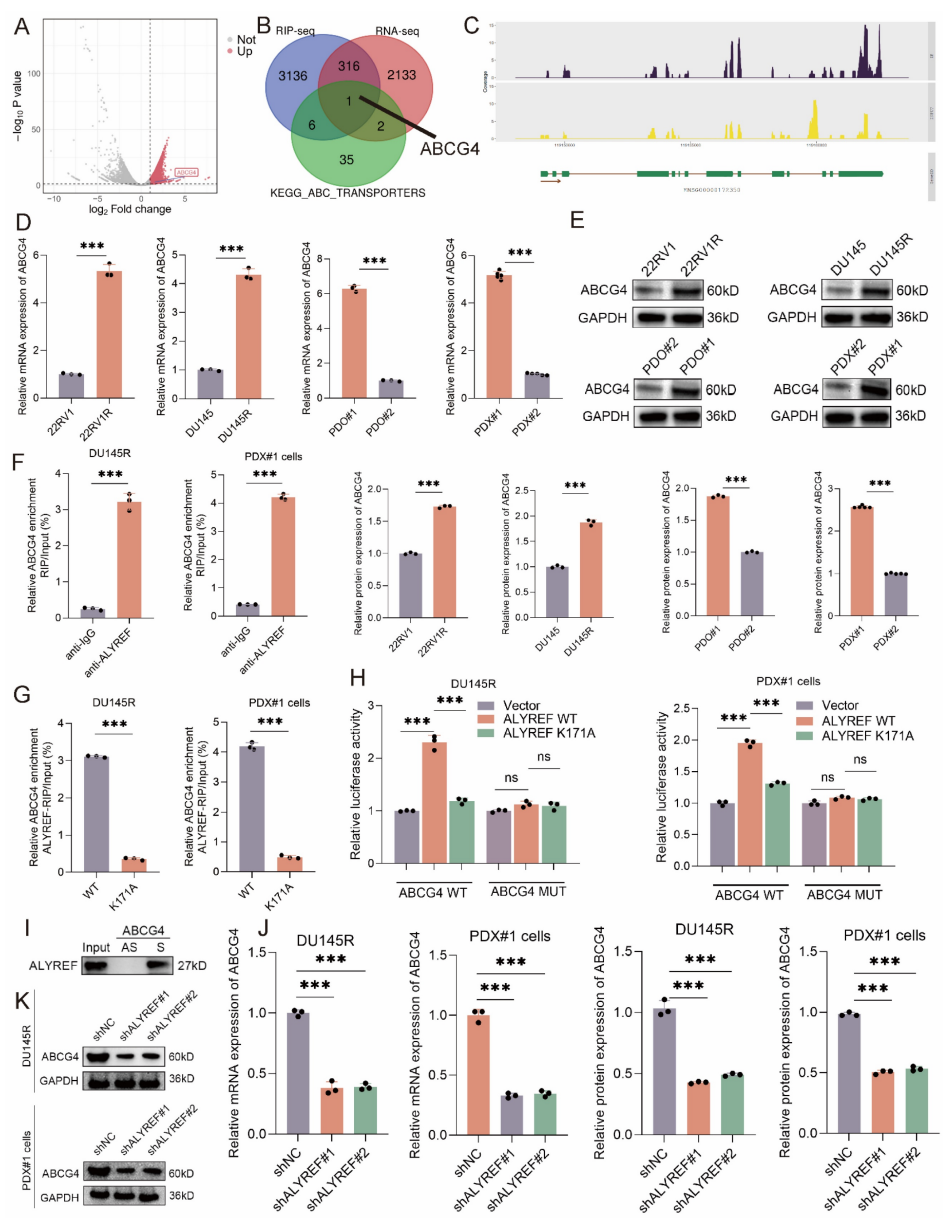
** ALYREF bound to the ABCG4 mRNA and upregulated ABCG4 expression.** Volcano map showing the potential binding mRNA of ALYREF identified by RIP-seq. (**B-C**) Overlap genes of RIP-seq (anti-Flag using lysates from 293T transfected with Flag-ALYREF), RNA-seq (shALYREF vs. shNC, PDO#1), and KEGG_ABC_TRANSPORTERS gene set. (**D-E**) The mRNA and protein expression levels of ABCG4 between DTX-resistant and DTX-sensitive CRPC cells (DU145R vs. DU145; 22RV1R vs. 22RV1), organoids (PDO#1 vs. PDO#2), PDXs (PDX#1 vs. PDX#2); unpaired two-tailed student's t-test. (**F**) Lysates from DTX-resistant CRPC cells (PDX#1 cells and DU145R) were subjected to RIP assays with antibodies against ALYREF or control IgG. three biological replicates were conducted; unpaired two-tailed student's t-test. (**G**) The effect of ALYREF K171A mutation (defect in m5C recognition) on the RIP enrichment for ABCG4 mRNA using lysates from DTX-resistant CRPC cells (PDX#1 cells and DU145R) subjected to RIP assays with antibodies against ALYREF or control IgG; three biological replicates were conducted; unpaired two-tailed student's t-test. (**H**) Reporter plasmids (ABCG4 WT or MUT) were co-transfected with plasmids for dual luciferase reporter assays. The relative luciferase activity was calculated. Three biological replicates were conducted; one-way ANOVA test. (**I**) Biotin-labeled RNA probes against full-length CDS (S) and antisense sequences (AS) were subjected to RNA pull-down assays (**J**) RT-qPCR showing the effect of ALYREF knockdown on the mRNA expression of ABCG4 in DU145R and PDX#1 cells; three biological replicates were conducted; one-way ANOVA test. (**K**) Western blot showing the effect of ALYREF knockdown on the protein expression of ABCG4 in DU145R and PDX#1 cells; three biological replicates were conducted; one-way ANOVA test. *P<0.05, **P<0.01, ***P<0.001.

To explore the downstream of ALYREF, we conducted ALYREF RIP-seq and RNA-seq (shALYREF vs. shNC, PDO#1). The volcano map showed the potential binding mRNA of ALYREF protein identified by RIP-seq (**Figure 5A**). The ABC efflux transporters have been proved as the major cause of DTX resistance^29^. The overlap gene of RIP-seq, RNA-seq, and KEGG_ABC_TRANSPORTERS gene set was merely ABCG4 (**Figure 5B-C**). RT-qPCR and western blot analysis revealed that ABCG4 mRNA and protein were significantly increased in DTX-resistant CRPC cells, organoids, PDXs compared with that in DTX-sensitive ones (DU145R vs. DU145; 22RV1R vs. 22RV1; PDO#1 vs. PDO#2; PDX#1 vs. PDX#2) (**Figure 5D-E**). Hence, high expression of ABCG4 resulting in DTX efflux might be one of the major causes of CRPC DTX resistance. The results of RIP-qPCR showed that ABCG4 mRNAs were enriched in complexes immunoprecipitated with antibodies against ALYREF compared to control IgG in DTX-resistant CRPC cells (DU145R and PDX#1 cells) (**Figure 5F**). As an m5C reader, the biological function of ALYREF depends on the m5C modification of target mRNAs. The results of RIP-qPCR showed that ABCG4 mRNAs were enriched in complexes immunoprecipitated with antibodies against ALYREF rather than ALYREF K171A (defect in m5C recognition) in DTX-resistant CRPC cells (DU145R and PDX#1 cells) (**Figure 5G**). Then, we used RNAm5cfinder tool and iRNA-m5C tool to predict the m5C sites of ABCG4, and the results revealed that the m5C site of ABCG4 might be C937. Our results of dual luciferase reporter assays using DTX-resistant CRPC cells (DU145R and PDX#1 cells) revealed that compared to the control, forced expression of wild-type ALYREF rather than ALYREF K171A mutation (defect in m5C recognition) enhanced the luciferase activity of wild-type ABCG4 but failed to increase that of mutant ABCG4 (**Figure 5H**). To confirm whether ALYREF directly binds to ABCG4 mRNA, we observed that a biotinylated probe binding to ABCG4 full-length CDS (S), but not to their antisense sequences (AS), pulled down the ALYREF proteins (**Figure 5I**). The results of RT-qPCR and western blot revealed that the knockdown of ALYREF could reduce the mRNA and protein expression levels of ABCG4 in DTX-resistant CRPC cells (DU145R and PDX#1 cells) (**Figure 5J-K**). The most common member of the ABC transporter family is ABCB1, and its role in DTX resistance in CRPC has also been reported^30^-^32^. We found that although ABCB1 mRNA and protein expression is upregulated in DTX-resistant CRPC; however, OTUB2/ALYREF axis seems to have no regulatory effect on ABCB1, suggesting that ABCB1 is not regulated by OTUB2/ALYREF axis. Other common ABC family members, which have been reported in DTX resistance of CRPC, included ABCC1, ABCC10, ABCG2, etc.^33^-^35^. Our preliminary results found that although ABCC1, ABCC10, and ABCG2 expression is upregulated in DTX-resistant CRPC; however, OTUB2/ALYREF axis seems to have no regulatory effect on ABCC1, ABCC10, and ABCG2, suggesting that ABCC1, ABCC10, and ABCG2 is not regulated by OTUB2/ALYREF axis (**Figure S4**).

**Figure 6:**
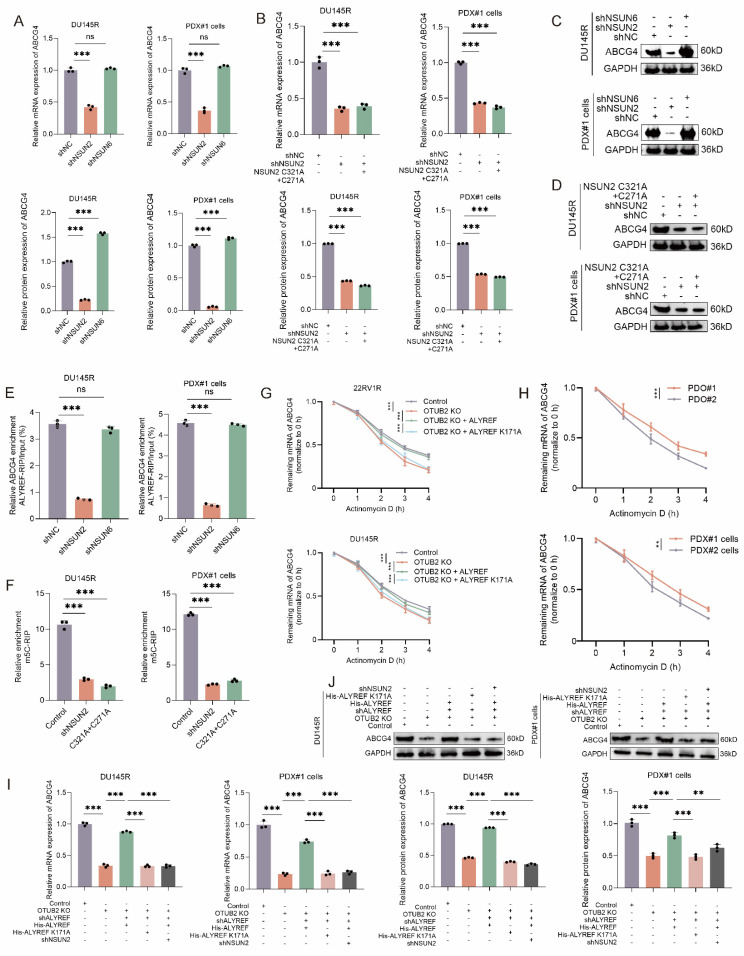
** ALYREF upregulated the mRNA stability of ABCG4 in a NSUN2-mediated m5C-dependent manner.** (**A**) RT-qPCR showing the effect of NSUN2 knockdown or NSUN6 knockdown on the expression of ABCG4 mRNA in DTX-resistant CRPC cells (PDX#1 cells and DU145R); three biological replicates were conducted; one-way ANOVA test. (**B**) RT-qPCR showing that the effect of NSUN2 knockdown on the expression of ABCG4 mRNA in DTX-resistant CRPC cells (PDX#1 cells and DU145R) could not be rescued by transduction of NSUN2 C321A+C271A mutation (defect in catalyzing m5C modification); three biological replicates were conducted; one-way ANOVA test. (**C**) Western blot showing the effect of NSUN2 knockdown or NSUN6 knockdown on the expression of ABCG4 protein in DTX-resistant CRPC cells (PDX#1 cells and DU145R); three biological replicates were conducted; one-way ANOVA test. (**D**) Western blot showing that the effect of NSUN2 knockdown on the expression of ABCG4 protein in DTX-resistant CRPC cells (PDX#1 cells and DU145R) could not be rescued by transduction of NSUN2 C321A+C271A mutation (defect in catalyzing m5C modification); three biological replicates were conducted; one-way ANOVA test. (**E**) The effect of NSUN2 knockdown or NSUN6 knockdown on the RIP enrichment for ABCG4 mRNA using lysates from DTX-resistant CRPC cells (PDX#1 cells and DU145R) subjected to RIP assays with antibodies against ALYREF or control IgG; three biological replicates were conducted; one-way ANOVA test. (**F**) Lysates from NSUN2 knockdown or mutation (C321A+C271A, defect in catalyzing m5C modification) DTX-resistant CRPC cells (PDX#1 cells and DU145R) were subjected to m5C-MeRIP assays using anti-m5C or anti-IgG antibodies. Transcripts of interest were detected by specific primers covering m5C sites; three biological replicates were conducted; one-way ANOVA test. (**G**) The effect of OTUB2/ALYREF axis on the residual mRNA levels of ABCG4 after termination of transcription via actinomycin D treatment in DTX-resistant CRPC cells (22RV1R and DU145R); three biological replicates were conducted; two-way repeated measures ANOVA test. (**H**) The residual mRNA levels of ABCG4 after termination of transcription via actinomycin D treatment between DTX-resistant and DTX-sensitive CRPC (PDX#1 cells vs. PDX#2 cells; PDO#1 vs. PDO#2); three biological replicates were conducted; two-way repeated measures ANOVA test. (**I**) RT-qPCR showing the effect of OTUB2/ALYREF axis on the mRNA expression levels of ABCG4 in DTX-resistant CRPC cells (PDX#1 cells and DU145R); three biological replicates were conducted; one-way ANOVA test. (**J**) Western blot showing the effect of OTUB2/ALYREF axis on the protein expression levels of ABCG4 in DTX-resistant CRPC cells (PDX#1 cells and DU145R); three biological replicates were conducted; one-way ANOVA test. *P<0.05, **P<0.01, ***P<0.001.

As NSUN2 and NSUN6 were previously well reported as m5C methyltransferases of mRNA, we used shRNA to knockdown their expression in DTX-resistant CRPC cells (DU145R and PDX#1 cells). The results of RT-qPCR and western blot revealed that the knockdown of NSUN2 rather than NSUN6 could reduce the mRNA and protein expression levels of ABCG4 in DTX-resistant CRPC cells (DU145R and PDX#1 cells). Besides, both NUSN2 knockdown and C321A+C271A mutation (defect in catalyzing m5C modification) could reduce the mRNA and protein expression levels of ABCG4 in DTX-resistant CRPC cells (DU145R and PDX#1 cells) (**Figure 6A-D**). The results of RIP-qPCR indicated that the enrichment ratio of ALYREF on ABCG4 mRNA was significantly reduced by NSUN2 knockdown, whereas no such effect was observed for NSUN6 knockdown (**Figure 6E**). The results of m5C-MeRIP assays using DTX-resistant CRPC cells (DU145R and PDX#1 cells) showed that NSUN2 knockdown or m5C methyltransferase dead mutation (C321A and C271A) significantly decreased the enrichment of transcripts detected by specific primers covering the predicted m5C sites of ABCG4 (C937) (**Figure 6F**). Moreover, we also found that the expression levels of NSUN2 mRNA and protein was significantly increased in DTX-resistant CRPC cells compared with that in DTX-sensitive ones (**Figure S5A-B**). To monitor the effects of OTUB2/ALYREF axis on the half-life of the ABCG4 mRNA, DTX-resistant CRPC cells (DU145R and 22RV1R) of indicated group were treated with the RNA synthesis inhibitor actinomycin D. The results suggested that OTUB2 knockout decreased the stability of ABCG4 mRNA and could be rescued by transduction of wide-type ALYREF rather than ALYREF K171A mutation (defect in m5C recognition). Besides, we found that the stability of ABCG4 mRNA was significantly increased in DTX-resistant CRPC cells (PDX#1 cells) and organoids (PDO#1) compared with that in DTX-sensitive CRPC cells (PDX#2 cells) and organoids (PDO#2) (**Figure 6G-H**). The results of RT-qPCR and western blot demonstrated that OTUB2 knockout decreased the mRNA and protein expression level of ABCG4 and could be rescued by transduction of wide-type ALYREF rather than ALYREF K171A mutation (defect in m5C recognition) in DTX-resistant CRPC cells (DU145R and PDX#1 cells) (**Figure 6I-J**). Theres results revealed that OTUB2/ALYREF axis upregulated the mRNA stability of ABCG4 in a NSUN2-mediated m5C-dependent manner.

### CAFs derived exosomal FOXD3-AS1 upregulates the expression of OTUB2 via sponging miR-5586-5p

**Figure 7:**
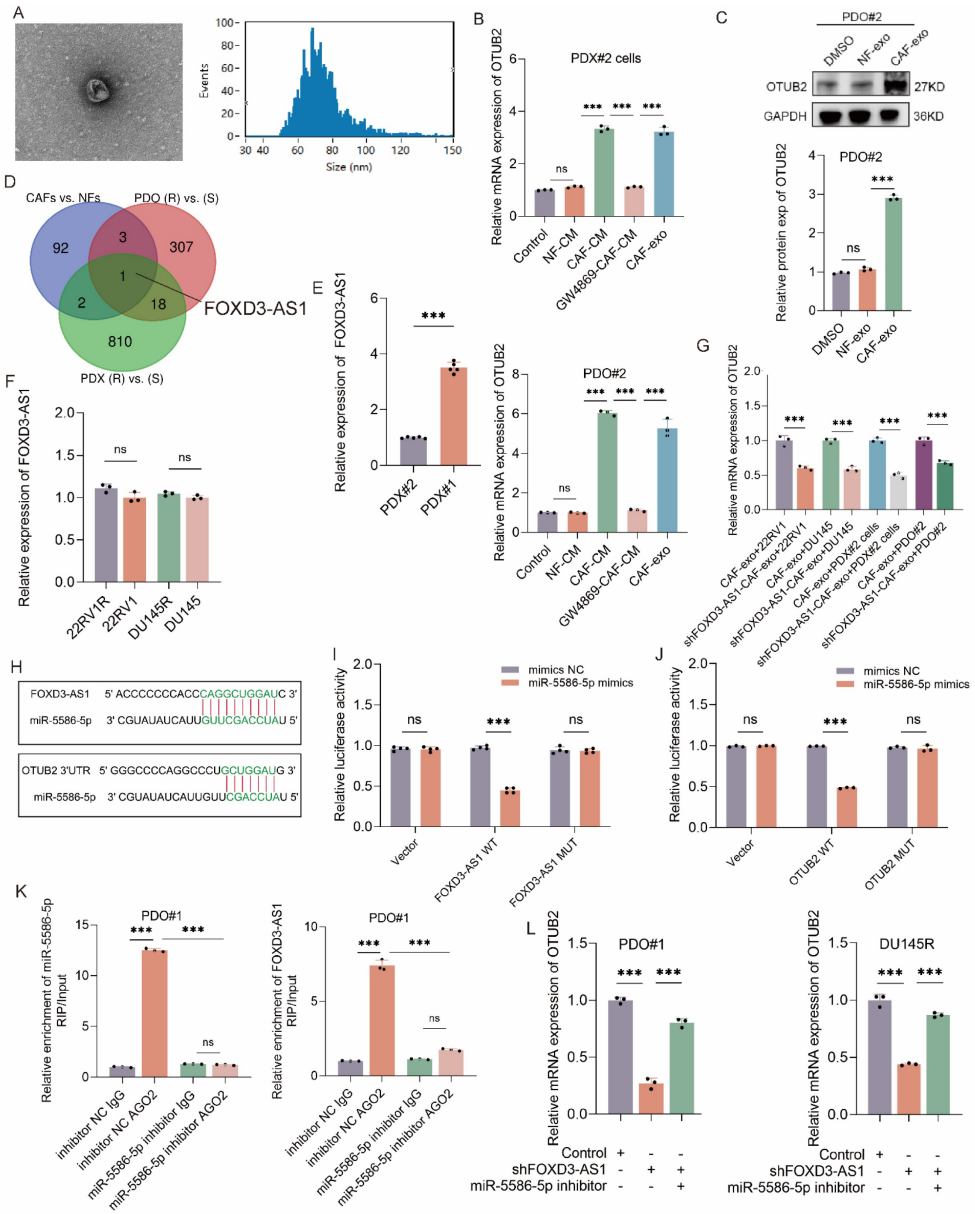
** CAF-derived exosomal FOXD3-AS1 upregulated the expression of OTUB2 via sponging miR-5586-5p.** (**A**) Electron microscopy and particle size diagram showing the successful extraction of exosome. (**B**) RT-qPCR showing the effect of CAF-derived exosomes on the expression of OTUB2 mRNA in DTX-sensitive CRPC cells (PDX#2 derived primary cells, PDX#2 cells) and organoids (PDO#2); three biological replicates were conducted; one-way ANOVA test. (**C**) Western blot showing the effect of CAF-derived exosomes treatment on the expression of OTUB2 protein in DTX-sensitive CRPC organoids (PDO#2); three biological replicates were conducted; one-way ANOVA test. (**D**) Venn diagram showing the intersection of differential expressed genes (CAFs exosomes vs. NFs exosomes; DTX-resistant vs. DTX-sensitive CRPC organoids; DTX-resistant vs. DTX-sensitive CRPC PDXs). (**E-F**) RT-qPCR indicating the differential expression level of FOXD3-AS1 between DTX-resistant and DTX-sensitive CRPC cells or PDXs; one-way ANOVA test. (**G**) RT-qPCR showing the effect of shFOXD3-AS1 CAFs-derived exosomes on the mRNA expression of OTUB2 in DTX-sensitive CRPC cells, including 22RV1, DU145, PDX#2 cells, and organoids (PDO#2); three biological replicates were conducted; one-way ANOVA test. (**H**) Combining site of FOXD3-AS1 and miR-5586-5p, OTUB2 and miR-5586-5p. (**I**) Parental (WT) and mutant FOXD3-AS1 were co-transfected with miR-5586-5p into 293T cells followed by dual luciferase assays; three biological replicates were conducted; unpaired two-tailed student's t-test. (**J**) Dual luciferase assays showing the effect of parental and mutant OTUB2 on the luciferase activity in 293T cells; three biological replicates were conducted; unpaired two-tailed student's t-test. (**K**) RIP assays were utilized to pulldown the endogenous RNA associated with AGO2 or IgG in DTX-resistant CRPC organoids (PDO#1) transfected with miR-5586-5p inhibitor or inhibitor NC; three biological replicates were conducted; one-way ANOVA test. (**L**) The results of RT‒qPCR showing the relative levels of OTUB2 in DTX-resistant CRPC organoids (PDO#1) and cells (DU145R) following different treatments; three biological replicates were conducted; one-way ANOVA test. *P<0.05, **P<0.01, ***P<0.001.

It has been reported that CAFs was implicated in DTX resistance of PCa, and exosome functioned as an important mediating factor^25^, ^36^. Here, we investigated whether CAFs impacted DTX resistance of CRPC via regulating OTUB2. In the RT-qPCR assay, NFs conditional medium (NFs-CM) culture did not affect the mRNA expression of OTUB2 in DTX-sensitive CRPC cells (22RV1, DU145, PDX#2 cells) and organoids (PDO#2) compared with the control group; CAFs conditional medium (CAFs-CM) culture (rather than GW4869-CAFs-CM culture) or CAFs-derived exosomes (CAFs-exo) treatment significantly upregulated the mRNA expression of OTUB2 compared with the control group or NFs-CM culture (**Figure 7A-B, Figure S5C**). In the western blot assay, NFs-exo treatment did not affect the protein expression of OTUB2 in DTX-sensitive CRPC cells (22RV1, DU145) and organoids (PDO#2) compared with the DMSO treatment; CAF-exo treatment could upregulate OTUB2 protein expression in DTX-sensitive CRPC cells (22RV1, DU145) and organoids (PDO#2) compared with DMSO or NF-exo treatment (**Figure 7C** and **Figure S5D**). Next, we explored how CAFs-derived exosomes regulated the expression level of OTUB2 mRNA and protein. Non-coding RNA has been reported as a vital component in exosome^37^. Hence, we conducted lncRNA RNA-seq in CAFs exosomes vs. NFs exosomes. The Venn diagram showing the intersection revealed that FOXD3-AS1 might be a major upregulated lncRNA in CAFs-derived exosomes, and might be one of key molecules of CAFs regulating DTX resistance of CRPC (**Figure 7D**). The results of RT-qPCR indicated that FOXD3-AS1 was upregulated in DTX-resistant CRPC PDXs compared with DTX-sensitive ones. However, the difference of expression level of FOXD3-AS1 between DTX-resistant and DTX-sensitive CRPC cells has no statistical significance (**Figure 7E-F**). Hence, the high expression of FOXD3-AS1 in CRPC PDXs might be originated from the tumor microenvironment, which further emphasized the role of CAFs.

Tumor-associated macrophage (TAM) also was reported to function in the DTX resistance of CRPC^38^, ^39^. To explore whether TAM affect the expression of OTUB2, we extracted the exosomes from M1 macrophage and TAM to treat DTX-sensitive CRPC cells (22RV1 and DU145), and found that TAM-derived exosomes did not affect the expression of OTUB2 mRNA and protein (**Figure S5E-F**). Hence, although there are studies reporting that TAM could also promote the DTX resistance of CRPC, TAM does not affect the DTX resistance of CRPC by influencing the OTUB2/ALYREF/ABCG4 axis. Besides, we found that CAF-derived exosome treatment significantly upregulated the expression of FOXD3-AS1 in DTX-sensitive CRPC cells (22RV1, DU145, PDX#2 cells) and organoids (PDO#2) compared with NF-derived exosome treatment (**Figure S5G**). These results revealed that CAFs derived exosome upregulated the expression level of FOXD3-AS1 and OTUB2 in DTX-sensitive CRPC.

Next, we explore whether and how CAFs derived exosome affects FOXD3-AS1 and OTUB2 expression in CRPC cells. The results of RT-qPCR showed that FOXD3-AS1 silencing in CRPC cells led to the downregulation of OTUB2 mRNA in DTX-resistant CRPC cells (22RV1R, DU145R, PDX#1 cells), and organoids (PDO#1) (**Figure S5H**). Besides, we silenced FOXD3-AS1 in CAFs and extracted exosomes to treat DTX-sensitive CRPC cells (22RV1, DU145, PDX#2 cells) and organoids (PDO#2). The results of RT-qPCR showed that exosome derived from CAFs transfected with shFOXD3-AS1 downregulated the expression levels of OTUB2 mRNA in DTX-sensitive CRPC cells (22RV1, DU145, PDX#2 cells) and organoids (PDO#2) compared with those transfected with shNC (**Figure 7G**). These results demonstrated that it was CAFs exosomal FOXD3-AS1 that upregulated the expression level of OTUB2 mRNA. We also found that there were predicted binding sites between miR-5586-5p and FOXD3-AS1, between miR-5586-5p and OTUB2 (**Figure 7H**). The results of RT‒qPCR showed that miR-5586-5p inhibitor upregulated the expression levels of OTUB2 mRNA in DTX-resistant CRPC cells (22RV1R, DU145R, PDX#1 cells) and organoids (PDO#1) compared with inhibitor NC (**Figure S5I**). To confirm the interaction of FOXD3-AS1 and miR-5586-5p, dual luciferase assays with reporters bearing FOXD3-AS1 (WT or MUT) at the putative miR-5586-5p binding site were conducted. We found that miR-5586-5p mimics significantly reduced the luciferase activity of FOXD3-AS1-WT, but not MUT (**Figure 7I**). Besides, miR-5586-5p mimics also remarkably decreased the luciferase activity of the OTUB2 reporter (**Figure 7J**). It has been widely reported that the lncRNA and miRNA interaction relies on an AGO2 complex^40^; therefore, we performed a AGO2-RIP assay to reveal that FOXD3-AS1 and miR-5586-5p was bound to the AGO2 ribonucleoprotein complex, which could be significantly inhibited by miR-5586-5p inhibitor (**Figure 7K and Figure S5J**). We then explored whether miR-5586-5p modulates FOXD3-AS1-mediated OTUB2 upregulation. After introducing the miR-5586-5p inhibitor into FOXD3-AS1 knockdown cells or PDOs, the addition of the miR-5586-5p inhibitor increased the OTUB2 mRNA level, which was downregulated following FOXD3-AS1 knockdown (**Figure 7L**). The *in vivo* results also revealed that miR-5586-5p inhibitor significantly increased the tumor weight and volume compared with the inhibitor NC, and could be rescued by knockdown of OTUB2 (**Figure S6A-C**). The results of RT-qPCR showed that miR-5586-5p inhibitor significantly increased the mRNA expression of OTUB2 and ABCG4 compared with the inhibitor NC, and could be rescued by knockdown of OTUB2 (**Figure S6D**). The results of western blot showed that miR-5586-5p inhibitor significantly increased the protein expression of OTUB2, ALREF, and ABCG4 compared with the inhibitor NC, and could be rescued by knockdown of OTUB2 (**Figure S6E**). These results demonstrated that miR-5586-5p is the upstream of OTUB2/ALYREF/ABCG4 axis and FOXD3-AS1 could regulated the expression level of OTUB2 by sponging miR-5586-5p.

### CAF exosomal FOXD3-AS1 facilitates the DTX resistance of CRPC via regulating OTUB2/ALYREF axis

**Figure 8:**
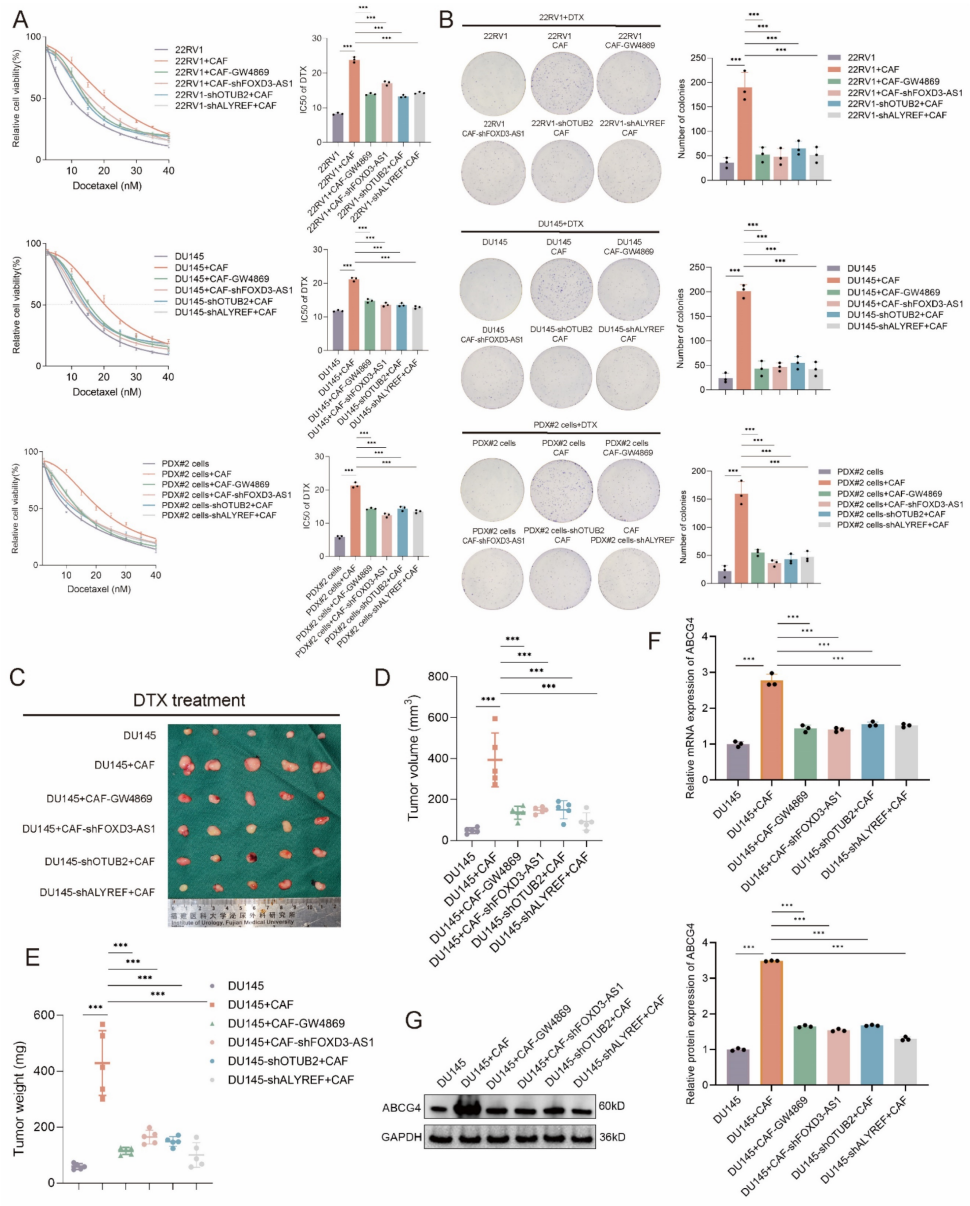
** CAF exosomal FOXD3-AS1 facilitates the DTX resistance of CRPC via regulating OTUB2/ALYREF axis *in vivo* and *vitro*.** (**A**) CCK8 toxicity assay showing the IC50 value of DTX of 22RV1, DU145, and PDX#2 derived primary cells (PDX#2 cells) of indicated group. The DTX-sensitive CRPC cells were transfected with shOTUB2 or shALYREF. The CAFs were treated with GW4869 or transfected with shFOXD3-AS1 and then the CAFs-conditioned medium (CAFs-CM) was used for culturing CRPC cells; three biological replicates were conducted; one-way ANOVA test (**B**) Representative images and quantitative results of cloning formation assay showing the cloning formation ability of 22RV1, DU145, and PDX#2 cells of indicated group. The DTX-sensitive CRPC cells were transfected with shOTUB2 or shALYREF. The CAFs were treated with GW4869 or transfected with shFOXD3-AS1 and then the CAFs-conditioned medium (CAFs-CM) was used for culturing CRPC cells; three biological replicates were conducted; one-way ANOVA test (**C-E**) Stable DU145 cells and CAFs of indicated groups were constructed, and then mixed and injected into the orthotopic prostate of male BALB/c nude mice. DTX was injected intraperitoneally (5 μg/g) every 3 days from day 12 onwards. Tumor images, weight and volume were obtained after dissection. (**F-G**) RT-qPCR and western blot showing the mRNA and protein expression of ABCG4 in the above orthotopic tumor of indicated groups; three biological replicates were conducted; one-way ANOVA test. *P<0.05, **P<0.01, ***P<0.001.

Then, we investigated whether CAF exosomal FOXD3-AS1 facilitated the DTX resistance of CRPC via regulating OTUB2/ALYREF axis. In the *in vitro* assay, the CAFs-conditioned medium (CAFs-CM) was used for culturing CRPC cells. According to the results of CCK8 toxicity assay, the IC50 value of DTX of 22RV1, DU145, PDX#2 cells was upregulated by culturing with CAFs-CM, and could be rescued by GW4869 treatment in CAFs, or shFOXD3-AS1 in CAFs, or shOTUB2 in CRPC cells, or shALYREF in CRPC cells (**Figure 8A**). According to the results of cloning formation assay under DTX treatment, cloning formation ability of 22RV1, DU145, PDX#2 cells was upregulated by culturing with CAFs-CM, and could be rescued by GW4869 treatment in CAFs, or shFOXD3-AS1 in CAFs, or shOTUB2 in CRPC cells, or shALYREF in CRPC cells (**Figure 8B**). The IC50 value of DTX-sensitive organoids (PDO#2) was upregulated by culturing with CAFs-CM, and could be rescued by GW4869 treatment in CAFs, or shFOXD3-AS1 in CAFs, or shOTUB2 in CRPC organoids, or shALYREF in CRPC organoids (**Figure S6F**). Stable DU145 cells and CAFs of indicated groups were constructed, and then mixed and injected into the orthotopic prostate of male BALB/c nude mice. DTX was injected intraperitoneally every 3 days from day 12 onwards. The growth of tumors derived from DU145 cells + CAFs was significantly faster than that in the DU145 cells group, and could be rescued by GW4869 treatment in CAFs, or shFOXD3-AS1 in CAFs, or shOTUB2 in CRPC cells, or shALYREF in CRPC cells. The weights and volumes of tumors derived from DU145 cells + CAFs were larger than that in the DU145 cells group, and could be rescued by GW4869 treatment in CAFs, or shFOXD3-AS1 in CAFs, or shOTUB2 in CRPC cells, or shALYREF in CRPC cells (**Figure 8C-E**). The results of RT-qPCR and western blot using xenograft tissues showed that the mRNA and protein expressions of ABCG4 in DU145 cells + CAFs group were higher than that in the DU145 cells group, and could be rescued by GW4869 treatment in CAFs, or shFOXD3-AS1 in CAFs, or shOTUB2 in CRPC cells, or shALYREF in CRPC cells (**Figure 8F-G**). The results of RT-qPCR showed that the expression of miR-5586-5p in 22RV1, DU145, PDX#2 cells, was downregulated by culturing with CAFs-CM, and could be rescued by GW4869 treatment in CAFs, or shFOXD3-AS1 in CAFs. Besides, the expression level of miR-5586-5p in the xenograft derived from DU145 cells + CAFs were decreased than that in the DU145 cells group, and could be rescued by GW4869 treatment in CAFs, or shFOXD3-AS1 in CAFs (**Figure S6G-I**). These results revealed that CAF exosomal FOXD3-AS1 facilitated the DTX resistance of CRPC via regulating OTUB2/ALYREF/ABCG4 axis *in vitro* and *in vivo*.

### Combination treatment with OTUB2-IN-1 reverses DTX resistance by suppressing the abundance of ABCG4

**Figure 9:**
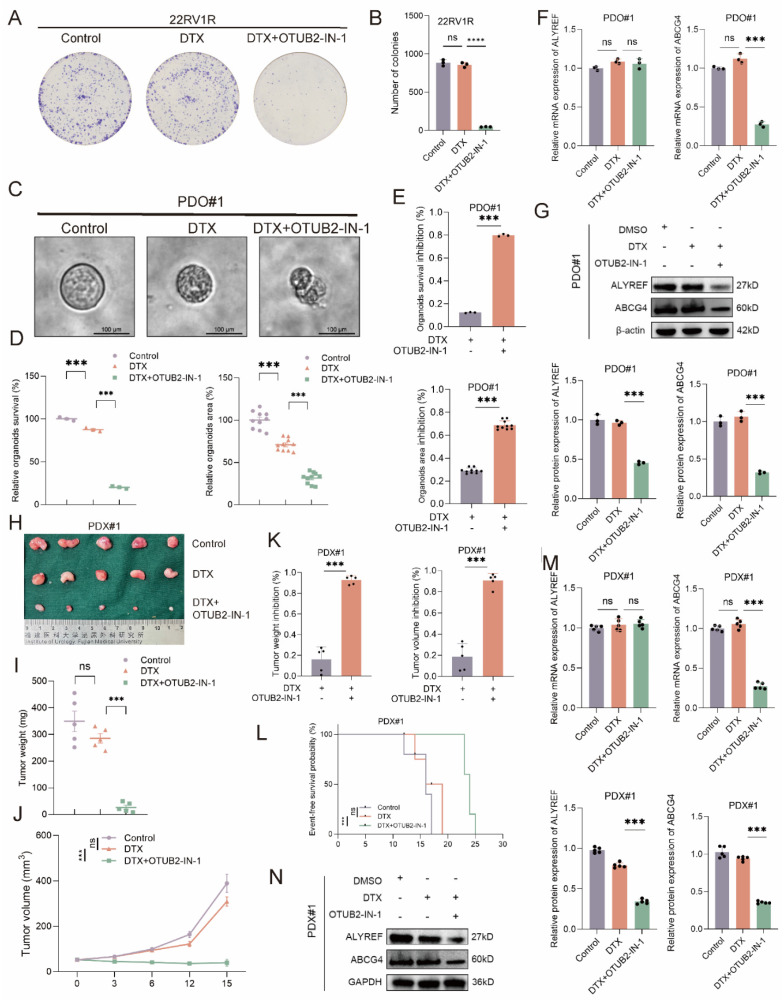
** Combination treatment with OTUB2-IN-1 reverses DTX resistance by suppressing the abundance of ABCG4. (A-B**) Representative images and quantitative results of cloning formation assay showing the effect of DTX combining with OTUB2-IN-1 on the cloning formation ability of 22RV1R; three biological replicates were conducted; one-way ANOVA test. (**C-E**) Representative bright-field images and the quantitative results of organoids area and viability of CRPC organoids showing the effect of DTX combining with OTUB2-IN-1 on the characteristics of DTX-resistant organoids (PDO#1). The effect of DTX combining with OTUB2-IN-1 on the organoids area and survival inhibition rate was also showed; one-way ANOVA test for D and unpaired two-tailed student's t-test for E. (**F-G**) The expression levels of ALYREF and ABCG4 were examined by RT-qPCR or western blot in DTX-resistant CRPC organoids (PDO#1) of indicated group; three biological replicates were conducted; one-way ANOVA test. (**H-J**) DTX-resistant CRPC PDXs (PDX#1) were transplanted into the flank of male BALB/c nude mice and treated with DTX and/or OTUB2-IN-1. Tumor volumes were measured every 3 days. Tumor images, weight and growth curves were obtained after dissection; one-way ANOVA test for I and two-way repeated measures ANOVA test for J. (**K**) The effect of DTX combining with OTUB2-IN-1 on the tumor weight and volume inhibition rate; unpaired two-tailed student's t-test. (**L**) The effect of DTX combining with OTUB2-IN-1 on the survival of tumor-bearing mice of PDX#1. The survival endpoint event was defined as a ≥ 20% decrease in body weight from the day of tumor inoculation; Kaplan-Meier analysis followed by Log-rank test. (**M-N**) The expression levels of ALYREF and ABCG4 were examined by RT-qPCR or western blot in DTX-resistant CRPC PDXs of indicated group; one-way ANOVA test. *P<0.05, **P<0.01, ***P<0.001.

We have established the vital role of OTUB2 in the development of DTX resistance of CRPC. Next, we explored whether the OTUB2 inhibitor sensitized the DTX chemotherapy of CRPC. OTUB2-IN-1 has been reported as an effective inhibitor of OTUB2 and they found that 10 μM OTUB2-IN-1 exhibited no obvious cytotoxicity on the viability of tumor cells^18^. We explored the non-specific cytotoxicity of OTUB2-IN-1 on the CRPC cells, and the results of CCK8 proliferation assay and colony formation assay revealed that the dose (10 μM) of OTUB2-IN-1 do not have non-specific toxicity to DU145R, 22RV1R, and PDX#1 cells (**Figure S7A-B**). Representative images and the quantitative results of cloning formation assay showed that OTUB2-IN-1 could sensitize the DTX chemotherapy of DTX-resistant CRPC cells and DTX combining with OTUB2-IN-1 has a synergistic anti-tumor effect (**Figure 9A-B, Figure S7C**). Representative bright-field images and the quantitative results of organoids area of DTX-resistant CRPC organoids (PDO#1) showed that OTUB2-IN-1 could sensitize the DTX chemotherapy of DTX-resistant CRPC organoids and DTX combining with OTUB2-IN-1 has a synergistic anti-tumor effect. The organoids survival of PDO#1 was significantly decreased after treating with DTX and OTUB2-IN-1. (**Figure 9C-E**). The results of RT-qPCR and western blot demonstrated that OTUB2-IN-1 inhibited the expression level of ALYREF protein and ABCG4 mRNA and protein rather than ALYREF mRNA in DTX-resistant CRPC organoids (PDO#1) (**Figure 9F-G**). DTX-resistant CRPC PDXs (PDX#1) were transplanted into the flank of male BALB/c nude mice and treated with DTX and/or OTUB2-IN-1. Tumor volumes were measured every 3 days. DTX was injected intraperitoneally every 3 days, starting after the tumor reaching 50-60 mm^3^. The tumor growth of PDX#1 in DTX combining with OTUB2-IN-1 group was significantly slower than that in the DTX group, and the tumor weights and volumes in DTX combining with OTUB2-IN-1 group were smaller than that in the DTX group. The tumor weight and volume inhibition rate in DTX combining with OTUB2-IN-1 group was significantly higher than that in the DTX group (**Figure 9H-K**). Besides, the survival rate of mice bearing PDX#1 of DTX combining with OTUB2-IN-1 group was significantly higher than that in the DTX group (**Figure 9L**). The results of RT-qPCR and western blot demonstrated that OTUB2-IN-1 affect the expression level of ALYREF protein and ABCG4 mRNA and protein rather than ALYREF mRNA in DTX-resistant CRPC PDXs (PDX#1) (**Figure 9M-N**). These results confirmed that OTUB2-IN-1 could sensitize the DTX chemotherapy of CRPC and DTX combining with OTUB2-IN-1 has a synergistic anti-tumor effect.

### Validation of the OTUB2/ALYREF/ABCG4 axis on the DTX resistance using CRPC PDXs and clinical samples

**Figure 10:**
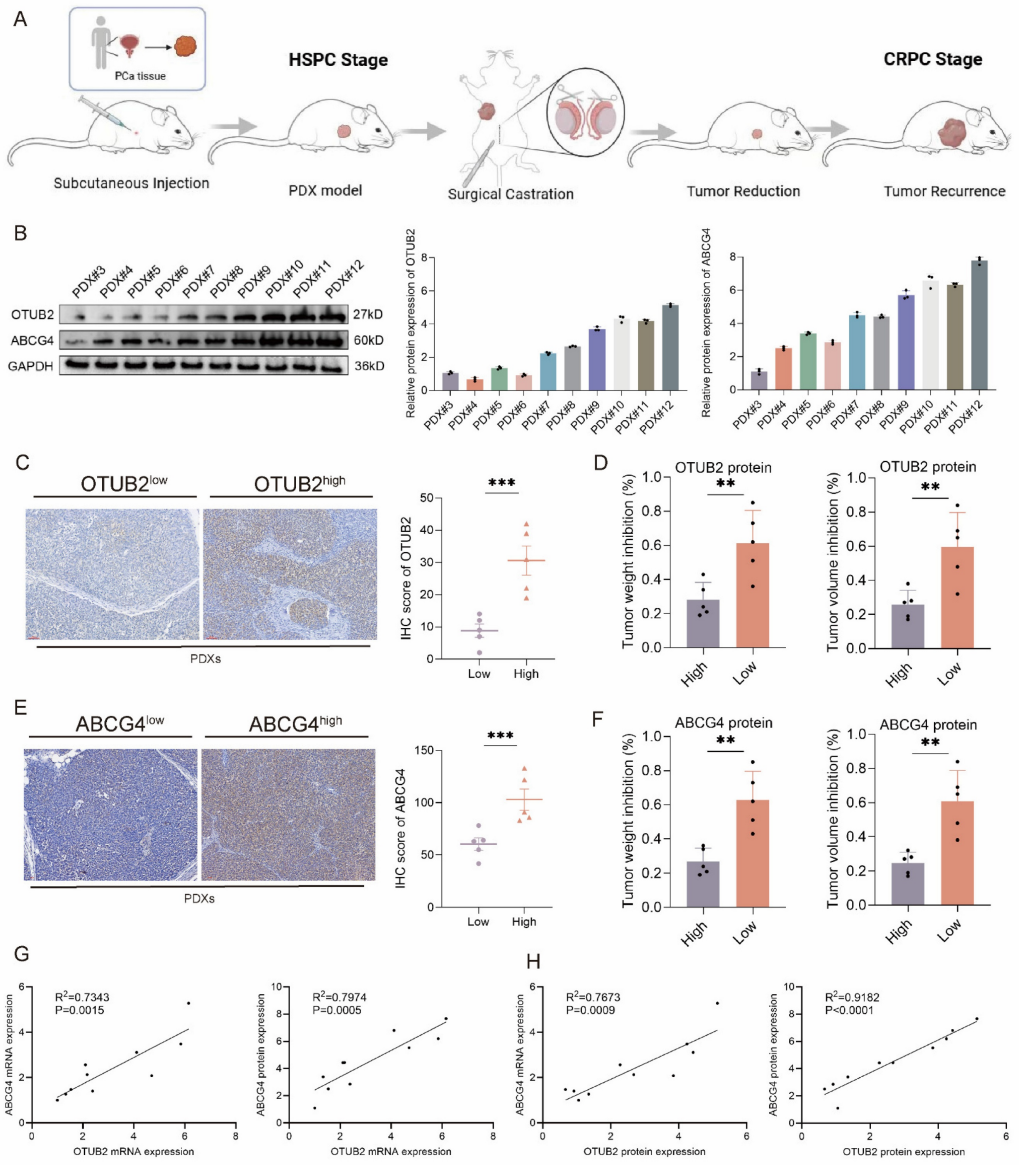
** Validation of the effect of OOTUB2 and ABCG4 expression on the DTX resistance using CRPC PDXs.** (**A**) Schematic diagram of constructing subcutaneous patient-derived xenograft (PDX) of CRPC via surgical castration of mice. (**B**) Western blot showing the protein expression level of OTUB2 and ABCG4 axis in 10 cases of CRPC PDXs; three biological replicates were conducted; one-way ANOVA test. (**C**) IHC staining showing the differential expression level of OTUB2 protein in OTUB2^high^ PDXs and OTUB2^low^ PDXs. (**D**) The effect of DTX on the tumor weight and volume inhibition rate between OTUB2^high^ PDXs and OTUB2^low^ PDXs; unpaired two-tailed student's t-test. (**E**) IHC staining showing the differential expression level of ABCG4 protein in ABCG4^high^ PDXs and ABCG4^low^ PDXs. (**F**) The effect of DTX on the tumor weight and volume inhibition between ABCG4^high^ PDXs and ABCG4^low^ PDXs; unpaired two-tailed student's t-test. (**G**) The expression association of OTUB2 mRNA with ABCG4 mRNA and protein among above 10 cases of CRPC PDXs. (**H**) The expression association of OTUB2 protein with ABCG4 mRNA and protein among above 10 cases of CRPC PDXs. *P<0.05, **P<0.01, ***P<0.001.

Next, to further validate the role of OTUB2 and ABCG4 in the CRPC DTX resistance, we constructed 10 cases of PDX models of HSPC using tumor tissues from radical prostatectomy, and then these 10 cases of HSPC PDXs were subsequently transitioned into CRPC PDXs models through surgical castration (**Figure 10A**). The results of western blot showed that the differential protein expression level of OTUB2 and ABCG4 axis in these 10 cases of CRPC PDXs (**Figure 10B**). Next, we divided these 10 cases of CRPC PDXs into OTUB2^high^ PDXs and OTUB2^low^ PDXs based on the median of the protein expression. The tumor weight and volume inhibition rate of DTX treatment in OTUB2^high^ PDXs were significantly decreased compared with that in OTUB2^low^ PDXs (**Figure 10C-D**). We also divided these 10 cases of CRPC PDXs into ABCG4^high^ PDXs and ABCG4^low^ PDXs based on the median of the protein expression. The tumor weight and volume inhibition rate of DTX treatment in ABCG4^high^ PDXs were significantly decreased compared with that in ABCG4^low^ PDXs (**Figure 10E-F**). We also found that there were positive associations of OTUB2 mRNA expression with ABCG4 mRNA and protein expression among above 10 cases of CRPC PDXs. There were positive associations of OTUB2 protein expression with ABCG4 mRNA and protein expression among above 10 cases of CRPC PDXs (**Figure 10G-H**). To further demonstrate the role of OTUB2/ALYREF/ABCG4 axis in the DTX resistance of CRPC, we enrolled 30 samples of CRPC patients receiving DTX chemotherapy and used RT-qPCR and IHC to determine the expression of OTUB2, ALYREF, and ABCG4. According to the medium value of expression levels, we divided all patients into high and low group. The results of survival analysis revealed that patients with high expression of OTUB2 mRNA, or OTUB2 protein, or ALYREF protein, or ABCG4 mRNA, or ABCG4 protein has a significantly decreased progression-free survival (PFS) compared with those with low expression (**Figure S8A-C**). We also found that there were positive associations of OTUB2 mRNA (or OTUB2 protein) expression with ALYREF protein (or ABCG4 mRNA, or ABCG4 protein expression) among these 30 cases of CRPC, and that there were positive associations of ALYREF protein expression with ABCG4 mRNA (or ABCG4 protein expression) among these 30 cases of CRPC (**Figure S8D-F**). These results confirmed that high expression of OTUB2 was associated with ABCG4-mediated DTX resistance of CRPC.

## Discussion

It has been reported that DTX resistance of CRPC had a strong correlation with aberrant activation of the AR signaling pathway, DNA damage repair, cancer stem cells and tumor microenvironment^8^-^10^, ^12^. However, the underlying mechanisms are still elusive and there is still a lack of effective molecular targets for improvement in DTX sensitivity. OTUB2, as member of the OTU family, was proved to be involved in chemotherapy resistance of various tumor types. Nan *et al.*^16^ revealed that epigenetic silencing of OTUB2 was a driver for mitochondrial metabolic reprogramming, promoting tumorigenesis and chemoresistance in ovarian cancer. It was demonstrated by Chang *et al.*^41^, that OTUB2 promoted the de-ubiquitination and phosphorylation of STAT1 and subsequently activated the transcription of CALML3, increasing sensitivity to chemotherapy in oral and esophageal squamous cell carcinomas. These findings highlighted OTUB2 exerted anti-chemoresistance effects. Conversely, Xu *et al.*^42^ reported that OTUB2 stabilized PGAM1 by mediating its K48-linked de-ubiquitination to prevent proteasomal degradation, promoting TMZ resistance of glioblastoma. In addition, multiple evidences have indicated that OTUB2 played a pro-tumor role in colorectal cancer^43^, gastric cancer^17^ and non-small cell lung cancer^44^. In our study, we discovered that OTUB2 was highly expressed in DTX-resistant CRPC and conferred the DTX resistance, which supported the notion of OTUB2 as a pro-chemoresistance factor. We revealed that high expression and the de-ubiquitination activity of tumoral OTUB2 promoted the DTX resistance of CRPC *in vitro* and *in vivo*. These findings emphasized the role of OTUB2 in modulating DTX resistance of CRPC.

In our study, the interacting protein ALYREF was identified by mass spectrometry after immunoprecipitation with OTUB2 antibody in DTX-resistant organoids. ALYREF, a member of the THO complex subunit family, functions as a pivotal RNA-binding protein and a specific reader for m5c modified RNA^45^, ^46^. It has been widely addressed that ALYREF is associated with tumor progression and chemoresistance. Huang *et al.*^47^ discovered that RNF31-mediated ALYREF cytoplasmic-nuclear shuttling enhanced the export of mRNAs encoding paclitaxel (PTX) resistance-related factors, inducing PTX resistance in triple-negative breast cancer. The result was found by Shi *et al.*^48^ that the activation of the NSUN2/ALYREF/MALAT1 signaling axis inhibited sorafenib-induced ferroptosis to drive sorafenib resistance in hepatocellular carcinoma. We revealed that OTUB2 conferred the docetaxel resistance of CRPC via removing the K48-linkage poly-ubiquitination and stabilizing ALYREF *in vitro* and *in vivo*. This finding provides a plausible mechanistic explanation for the pro-chemoresistance function of OTUB2 in CRPC.

Drug efflux mediated by ABC transporters, especially ABCB1 (MDR1/P-gp), is a well-characterized classic multidrug resistance mechanism^49^. The primary physiological function of ABCG4, another key member of the ATP-binding cassette (ABC) transporter family, is to regulate the transport of cholesterol and sterols, participating in maintaining intracellular lipid homeostasis^50^. Recently, ABCG4 was also reported to reduce intracellular drug concentration by mediating drug efflux, thereby inducing chemoresistance in various cancers^50^. Chen *et al.*^51^ demonstrated that tumor-associated macrophage-derived CCL22 upregulated the expression of ABCG4, ABCG3, and ABCG5 via the DKG/NF-κB signaling pathway, reducing intracellular drug accumulation and promoting chemoresistance in esophageal squamous cell carcinoma. Furthermore, clinical evidences have indicated that high ABCG4 expression was associated with poor prognosis in non-small cell lung cancer patients receiving cisplatin-based chemotherapy^52^; while low ABCG4 expression correlated with better chemotherapeutic response in breast cancer^53^. Drug efflux mediated by ABC transporters is a well-established mechanism underlying DTX resistance and the role of four ABC transporter family, ABCB1, ABCC1, ABCC10, and ABCG2 has also been reported in the development of DTX resistance of CRPC^30^, ^31^, ^33^, ^34^; however, the specific role of ABCG4 on DTX resistance in CRPC remains unclear. In our study, integrative analysis of ALYREF RIP-seq, RNA-seq, and the KEGG_ABC_TRANSPORTERS gene set identified ABCG4 as a potential downstream target of ALYREF. Using DTX-resistant CRPC cells, organoids and PDXs, we revealed that ALYREF stabilized ABCG4 mRNA in an m5C-dependent manner, resulting in ABCG4 upregulation and consequently promoting DTX resistance in CRPC. Furthermore, the OTUB2 inhibitor OTUB2-IN-1 potently suppressed ABCG4 expression in DTX-resistant CRPC organoids and PDX models, which furtherly supports the involvement of the OTUB2/ALYREF/ABCG4 signaling axis in DTX resistance of CRPC. These findings provided the novel mechanistic insight into the role of ABCG4 in driving DTX resistance of CRPC and elucidate its upstream regulatory network.

CAFs, being one of the most abundant stromal components in the TME, remodel extracellular matrix, suppress tumor immune response, and aggravate tumor chemoresistance, by intercellular communication^54^-^56^. Exosomes, microscale vesicles secreted from a variety of cells, with high stability and biocompatibility, are recognized as the one of the most common modalities for intercellular communication^57^, ^58^. Previous studies have indicated that CAFs-derived exosomes participated in the regulation of PCa progression and chemoresistance^59^-^61^. Xiong, *et al.* revealed that CAFs plays an important role in regulating mitochondrial metabolism to inhibit PCa chemosensitivity via ANGPTL4/IQGAP1 axis^36^. Zhao, *et al.* found that miR-432-5p from CAFs inhibits ferroptosis and promotes chemoresistance of PCa via targeting CHAC1^25^. Among the diverse cargoes packaged within exosomes, lncRNAs represent prominent molecules known to exert significant regulatory functions^62^-^64^. Qu *et al.*^65^ found that CAFs-derived exosomal DACT3-AS1 is a suppressive regulator in oxaliplatin resistance of gastric cancer. The result was revealed by Deng *et al.*^66^, that lncRNA CCAL transferred from CAFs by exosomes promoted chemoresistance of colorectal cancer cells. Luo *et al.*^67^ demonstrated that exosomal LINC00355 derived from CAFs promoted resistance to cisplatin by regulating miR-34b-5p/ABCB1 axis in bladder cancer cells. In our results, we found that CAFs-derived exosomes upregulated the mRNA and protein expression of OTUB2 in DTX-sensitive CRPC cells and organoids. The CAFs derived exosomal FOXD3-AS1 was finally identified as a potential regulator for expression level of OTUB2 in DTX-sensitive CRPC. lncRNAs function as ceRNAs by competitively sponging miRNAs, modulating expression of target mRNAs. Pan *et al.*^68^ found that lncRNA JPX upregulated Twist1 by competitively sponging miR-33a-5p and subsequently induced EMT and lung cancer cell invasion. Zhang *et al.*^69^ discovered that lncRNA NEAT1 regulated MIOX expression by sponging miR-362-3p as a ceRNA, promoting ferroptosis. In our study, it was revealed that the exosomal FOXD3-AS1 transfers from CAFs to CRPC cells, up-regulating the RNA and protein expression of OTUB2 via sponging miR-5586-5p. Moreover, this mechanism facilitates the DTX resistance of CRPC via OTUB2/ALYREF axis.

Our study firstly revealed that OTUB2 functioned as a novel driver for DTX resistance of CRPC by deubiquitinating ALYREF. The m5C reader ALYREF upregulated the mRNA stability of ABCG4 to facilitate ATP-dependent docetaxel drug efflux. Additionally, it was emphasized that the role of CAFs-derived exosomal FOXD3-AS1 in modulating DTX resistance of CRPC via sponging miR-5586-5p. OTUB2 may be a potential target for DTX sensitization of CRPC. However, there are limitations of this study to note. Firstly, the role of OTUB2 in the DTX resistance required validation by conditional knockout mice. Secondly, we applied the organoids and PDX models to retain the biological characteristics of the CRPC, but models still cannot fully simulate tumor microenvironment. Thirdly, the clinical application of OTUB2 inhibitor need further verification. Fourthly, AR is central to prostate cancer development, and the CRPC could be divided into AR-positive and AR-negative subpopulation. There have been researches reporting that AR in prostate cancer could be ubiquitinated^70^, ^71^. Although our results demonstrated that OTUB2/ALYREF axis conferred the docetaxel resistance of CRPC via ABCG4, we did not investigate whether the de-ubiquitinase OTUB2 could directly regulate the ubiquitination of AR in CRPC cells. This issue required further exploration in future. Finally, the models used in this study could not fully assess the interaction between CAFs and immune cells, which deserves more in-depth exploration in the future.

## Conclusion

OTUB2 is a driver of DTX resistance of CRPC. Mechanistically, OTUB2 leads to the increased expression of ALYREF protein by regulating K48 ubiquitination, and then the m5C reader ALYREF upregulated the mRNA stability of ABCG4 to facilitate docetaxel drug efflux. The exosomal FOXD3-AS1 transfers from CAFs to CRPC cells, up-regulating the mRNA and protein expression of OTUB2 via sponging miR-5586-5p. OTUB2 inhibitor (OTUB2-IN-1) could sensitize the DTX chemotherapy of DTX-resistant CRPC, and DTX combining with OTUB2-IN-1 has a synergistic anti-tumor effect. Our findings emphasized the role of OTUB2 and CAFs in modulating docetaxel resistance of CRPC.

## Supplementary Material

Supplementary figures and tables.

## Data Availability

The data that support the findings of this study are available from the corresponding author upon reasonable request.
