# Supplementary material for: OTUB2/ALYREF axis modulates the docetaxel resistance of castration-resistant prostate cancer via upregulating ABCG4-mediated drug efflux
Source: Int J Biol Sci. 2026 Mar 17;22(7):3342–66. doi: 10.7150/ijbs.126319 (PMC13085892; doi:10.7150/ijbs.126319)
Supplement: Supplementary file 1 — Supplementary figures and tables. [file ijbsv22p3342s1.pdf]

Supplementary Materials

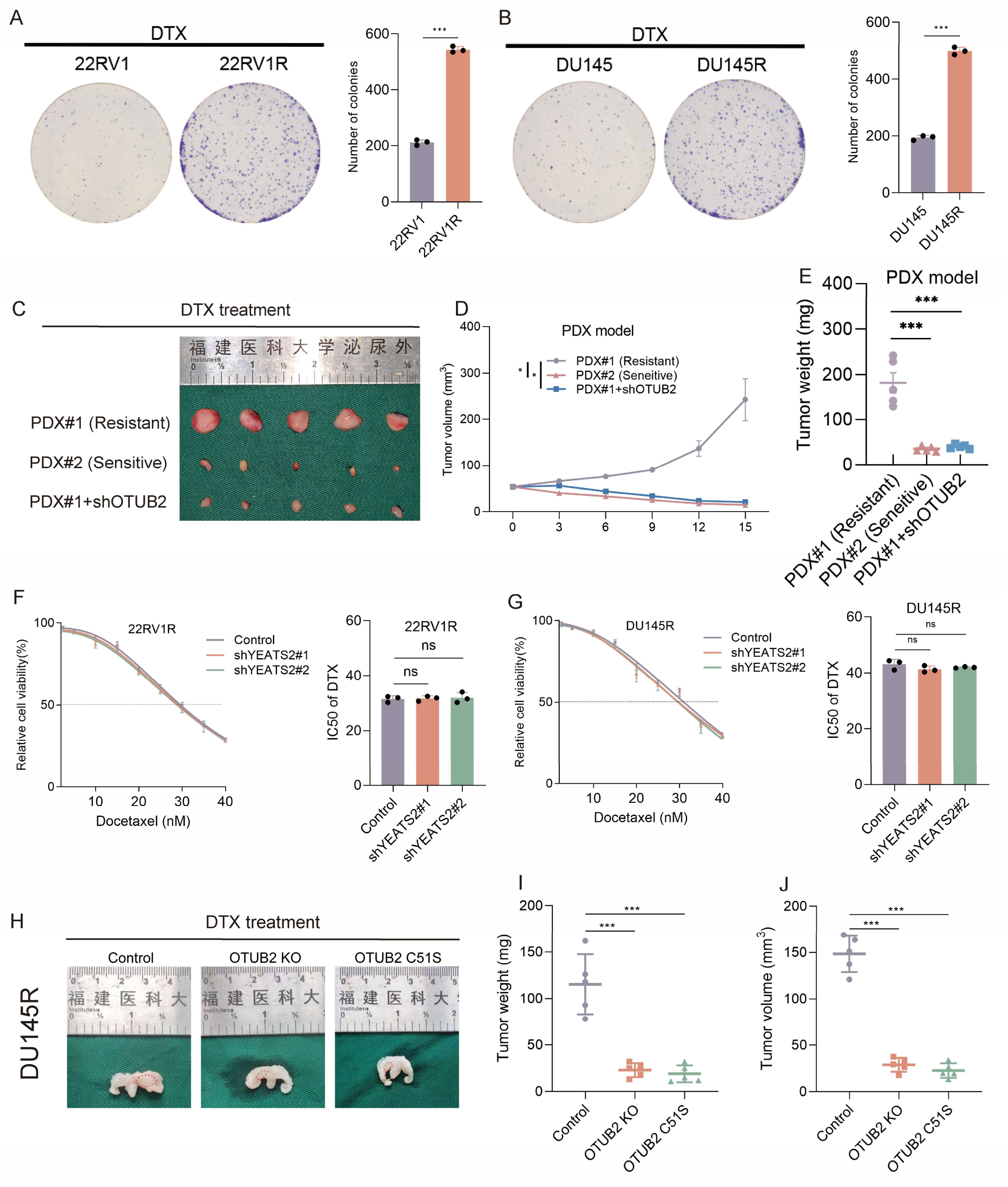

Figure S1. Construction of DTX-resistant CRPC cells and validation of the role of OTUB2 and YEATS2 on the DTX resistance of CRPC.

(**A-B**) Representative images and quantitative results of cloning formation assay showing the difference of DTX-resistant and DTX-sensitive CRPC cells (22RV1R vs. 22RV1; DU145R vs, DU145); three biological replicates were conducted; unpaired two-tailed student's t-test. (**C-E**) CRPC PDXs from two cases of CRPC patients were transplanted into the flank of male BALB/c nude mice. The lentivirus carrying OTUB2 shRNA was injected into CRPC PDX#1 tumor. Tumor volumes were measured every 3 days. Tumor images, weight and growth curves were obtained after dissection. DTX was injected intraperitoneally every 3 days; two-way repeated measures ANOVA test for D and one-way ANOVA test for E. (**F-G**) The effect of YEATS2 knockdown on the DTX IC50 of CRPC cells (22RV1R and DU145R); three biological replicates were conducted; one-way ANOVA test. (**H-J**) Stable DU145R

cells of indicated groups were injected into the orthotopic prostate of male BALB/c nude mice. DTX was injected intraperitoneally every 3 days from day 12 onwards. Tumor images, weight and volume were obtained after dissection. **P*<0.05, ***P*<0.01, ****P*<0.001.

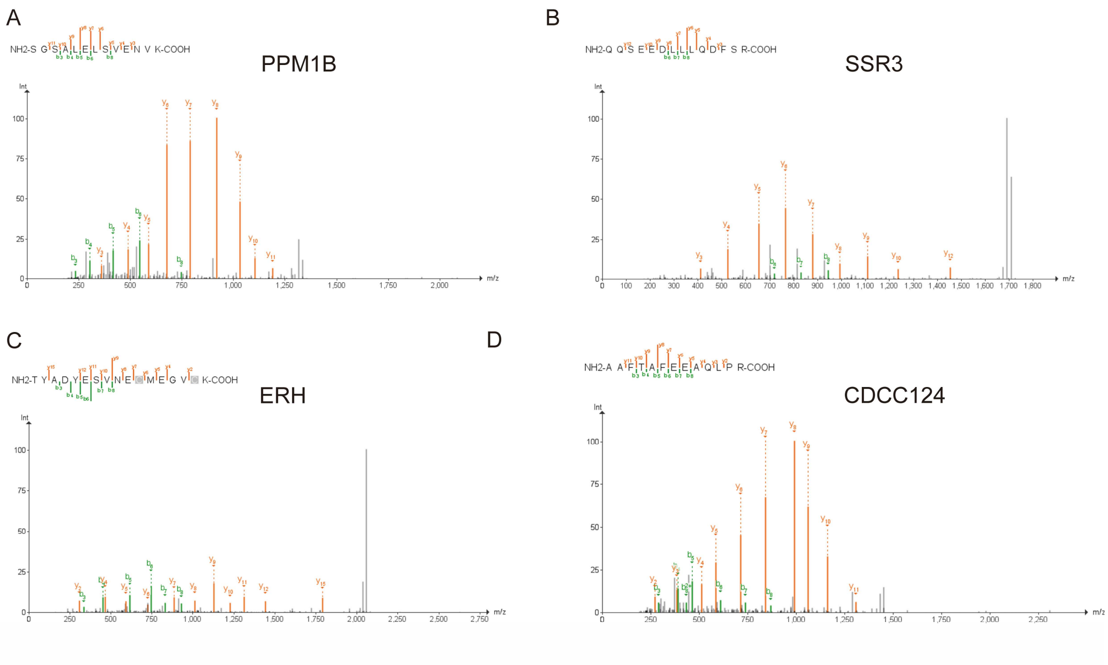

Figure S2. The secondary diagram of MS of PPM1B (A), SSR3 (B), ERH (C), and CCDC124 (D).

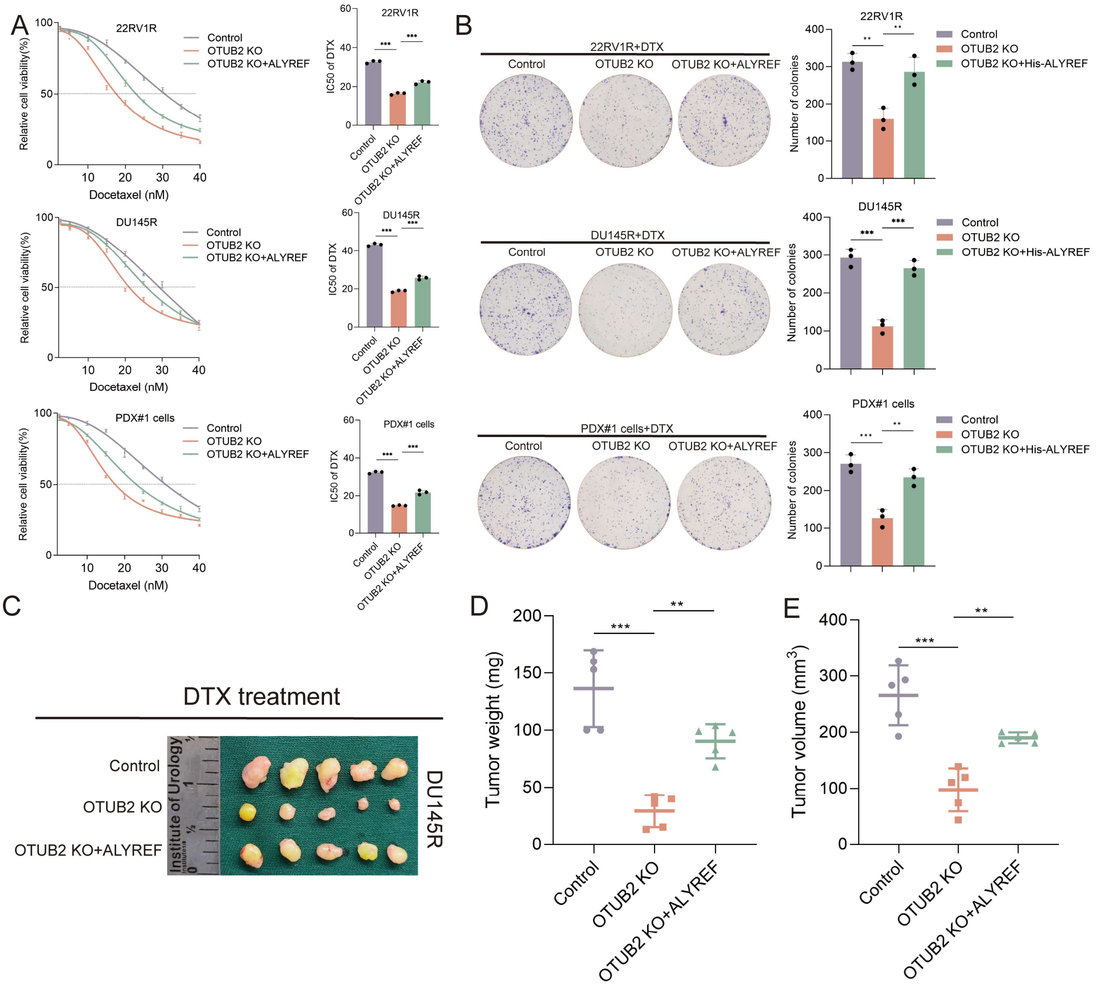

Figure S3. OTUB2 promoted the DTX resistance of CRPC via upregulating ALYREF *in vivo* and *in vitro*.

(**A**) CCK8 toxicity assay showed that the effect of OTUB2 knockout on the IC50 value of DTX of 22RV1R, DU145R, and PDX#1 cells could be rescued by transducing His-ALYREF; three biological replicates were conducted; one-way ANOVA test. (**B**) Representative images and quantitative results of cloning formation assay showing the effect of OTUB2 knockout on the cloning formation ability of 22RV1R, DU145R, and PDX#1 cells could be rescued by transducing His-ALYREF; three biological replicates were conducted; one-way ANOVA test. (**C-E**) Stable DU145R cells of indicated groups were injected into the orthotopic prostate of male BALB/c nude mice. DTX was injected intraperitoneally every 3 days from day 12 onwards. Tumor images, weight and volume were obtained at after dissection. One-way ANOVA test. *P<0.05, **P<0.01, ***P<0.001.

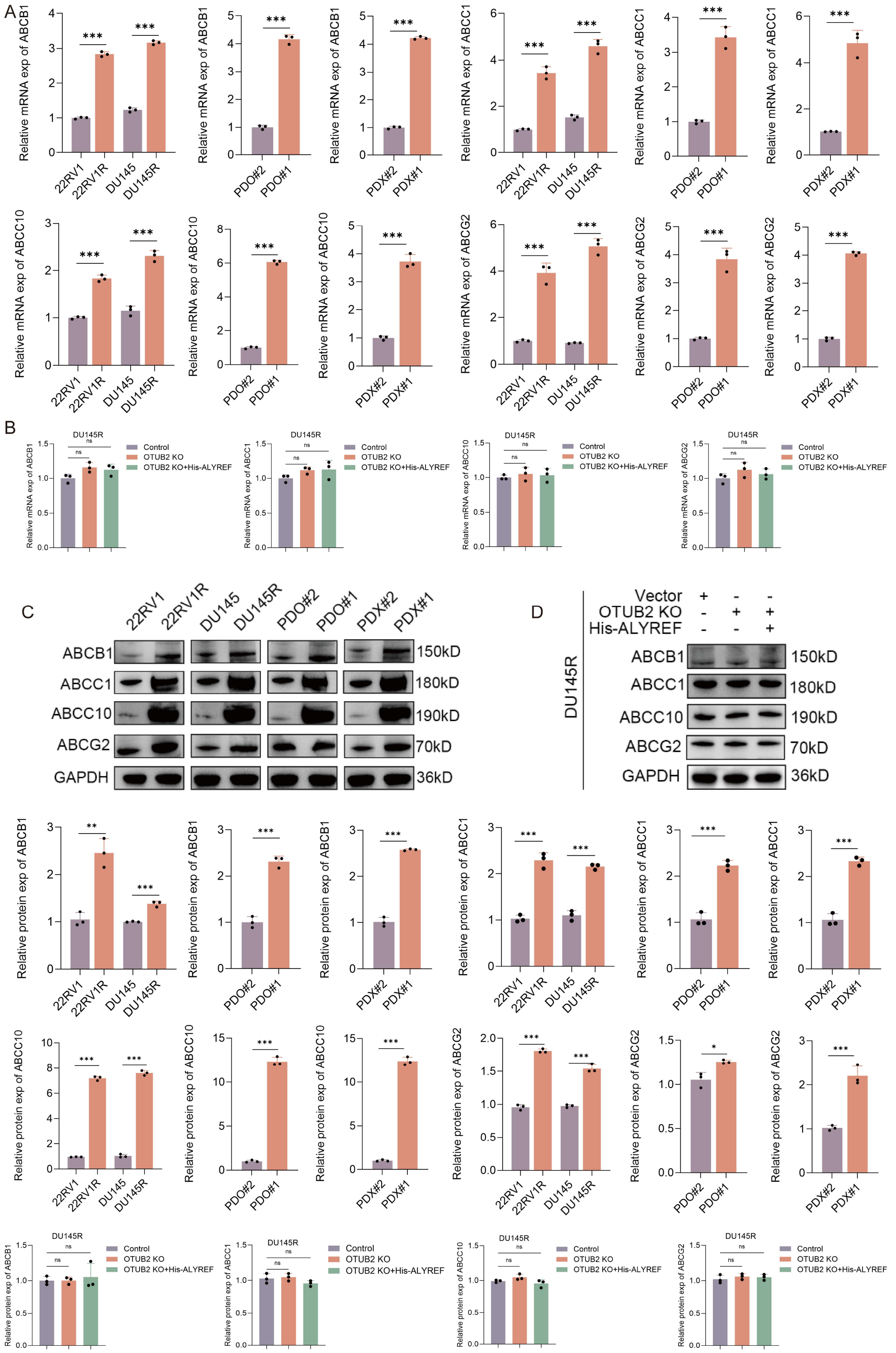

Figure S4. The role of ABCB1, ABCC1, ABCC10, and ABCG2 in the development of DTX resistance of CRPC cells.

(**A**) The results of RT-qPCR showing the difference of the mRNA expression of ABCB1, ABCC1,

ABCC10, and ABCG2 between DTX-resistant and DTX-sensitive CRPC cells (22RV1R vs. 22RV1; DU145R vs. DU145), organoids (PDO#1 vs. PDO#2), and PDXs (PDX#1 vs. PDX#2); three biological replicates were conducted; unpaired two-tailed student's t-test. (**B**) The results of RT-qPCR showing the effect of OTUB2/ALYREF axis on the mRNA expression of ABCB1, ABCC1, ABCC10, and ABCG2 in DTX-resistant CRPC cells; three biological replicates were conducted; one-way ANOVA test. (**C**) The results of western blot showing the difference of the protein expression of ABCB1, ABCC1, ABCC10, and ABCG2 between DTX-resistant and DTX-sensitive CRPC cells (22RV1R vs. 22RV1; DU145R vs. DU145), organoids (PDO#1 vs. PDO#2), and PDXs (PDX#1 vs. PDX#2); three biological replicates were conducted; unpaired two-tailed student's t-test. (**D**) The results of western blot showing the effect of OTUB2/ALYREF axis on the protein expression of ABCB1, ABCC1, ABCC10, and ABCG2 in DTX-resistant CRPC cells; three biological replicates were conducted; one-way ANOVA test. *$P<0.05$, **$P<0.01$, ***$P<0.001$.

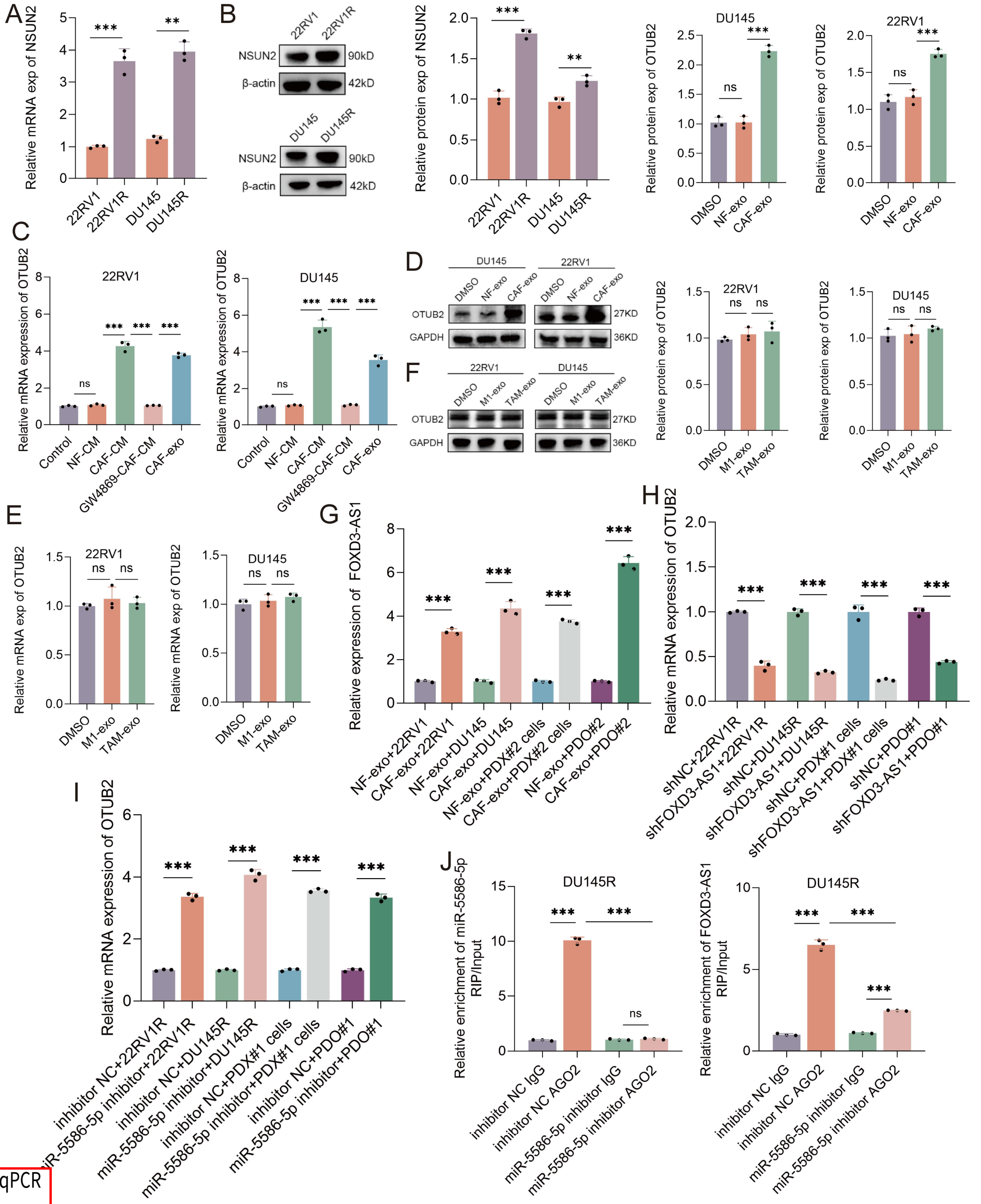

it should be "RT-qPCR and western blot showing the difference of mRNA and protein expression of NSUN2"

[illegible]re S5. Validation of the differential expression of NSUN2 and CAF-derived exosomal [illegible]D3-AS1 upregulating the expression of OTUB2 via sponging miR-5586-5p.

(**A-B**) The results of RT-qPCR showing the difference of the mRNA expression of NSUN2 between DTX-resistant and DTX-sensitive CRPC cells (22RV1R vs. 22RV1; DU145R vs. DU145); three biological replicates were conducted; unpaired two-tailed student's t-test. (**C**) RT-qPCR showing the effect of CAF-derived exosomes on the expression of OTUB2 mRNA in DTX-sensitive CRPC cells (22RV1 and DU145); three biological replicates were conducted; one-way ANOVA test. (**D**) Western blot showing the effect of CAF-derived exosomes on the expression of OTUB2 protein in DTX-sensitive CRPC cells (22RV1 and DU145); three biological replicates were conducted; one-way ANOVA test. (**E**) RT-qPCR showing the effect of TAM-derived exosomes on the expression of

OTUB2 mRNA in DTX-sensitive CRPC cells (22RV1 and DU145); three biological replicates were conducted; one-way ANOVA test. (**F**) Western blot showing the effect of TAM-derived exosomes on the expression of OTUB2 protein in DTX-sensitive CRPC cells (22RV1 and DU145); three biological replicates were conducted; one-way ANOVA test. (**G**) RT-qPCR showing the effect of CAF or NFs-derived exosomes treatment on the expression of FOXD3-AS1 in DTX-sensitive CRPC cells (22RV1, DU145, and PDX#2 cells), and organoids (PDO#2); three biological replicates were conducted; unpaired two-tailed student's t-test. (**H**) RT-qPCR showing the effect of FOXD3-AS1 silencing in CRPC cells on the mRNA expression of OTUB2 in DTX-resistant CRPC cells (22RV1R, DU145R, PDX#1 cells), and organoids (PDO#1); three biological replicates were conducted; unpaired two-tailed student's t-test. (**I**) RT–qPCR measuring the relative expression of OTUB2 mRNA in DTX-resistant CRPC cells (22RV1R, DU145R, PDX#1 cells), and organoids (PDO#1) followed by transfection with miR-5586-5p inhibitors; three biological replicates were conducted; unpaired two-tailed student's t-test. (**J**) RIP assays were utilized to measure the endogenous RNA associated with AGO2 in DTX-resistant CRPC cells (DU145R) transfected with miR-5586-5p inhibitor or inhibitor NC; three biological replicates were conducted; one-way ANOVA test. *$P<0.05$, **$P<0.01$, ***$P<0.001$.

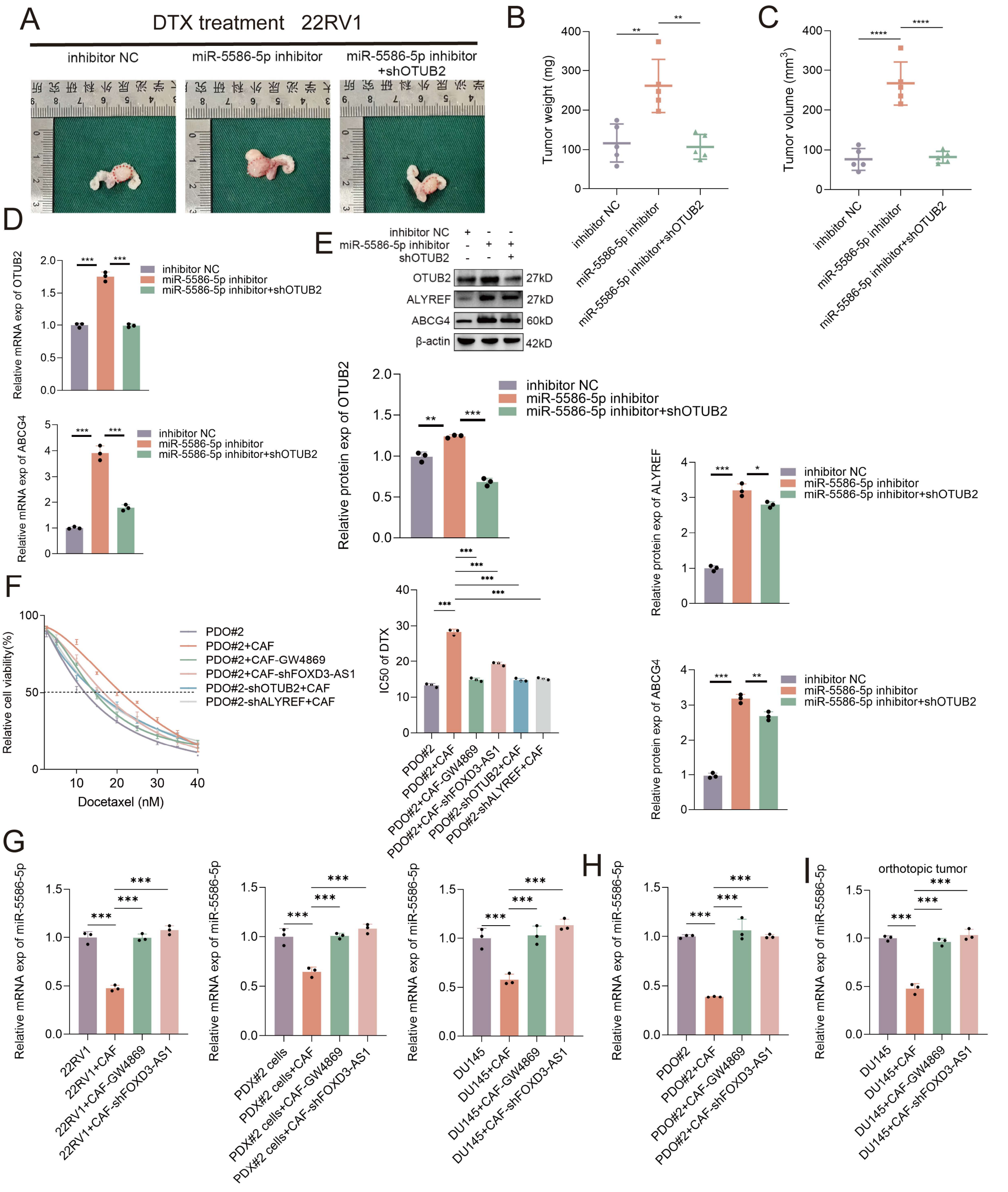

Figure S6. The role of miR-5586-5p in the DTX resistance and CAF exosomal FOXD3-AS1 facilitates the DTX resistance via regulating OTUB2/ALYREF axis.

(**A-C**) 22RV1 cells of indicated group (inhibitor NC, miR-5586-5p inhibitor, miR-5586-5p inhibitor+shOUTB2) were injected into the orthotopic prostate of male BALB/c nude mice. DTX was injected intraperitoneally every 3 days from day 12 onwards. Tumor images, weight and volume were obtained after dissection. One-way ANOVA test. (**D**) RT-qPCR showing the difference of the expression of OUTB2 mRNA and ABCG4 mRNA in the orthotopic xenografts of indicated group (inhibitor NC, miR-5586-5p inhibitor, miR-5586-5p inhibitor+shOUTB2); three biological replicates were conducted; one-way ANOVA test. (**E**) Western blot showing the difference of the expression of OTUB2 protein, ALYREF protein, ABCG4 protein in the orthotopic xenografts of indicated group (inhibitor NC, miR-5586-5p inhibitor, miR-5586-5p inhibitor+shOUTB2); three biological replicates

were conducted; one-way ANOVA test. (**F**) The IC50 value of DTX of DTX-sensitive CRPC organoids (PDO#2) of indicated group. The DTX-sensitive CRPC organoids (PDO#2) were transfected with shOTUB2 or shALYREF. The CAFs were treated with GW4869 or transfected with shFOXD3-AS1. (**G**) The results of RT-qPCR showing the effect of CAFs-CM on the expression of it should be "I" [illegible]6-5p in 22RV1, DU145, PDX#2 cells, and could be rescued by GW4869 treatment in CAFs, or shFOXD3-AS1 in CAFs. (**H**) The results of RT-qPCR showing the effect of CAFs-CM on the expression of miR-5586-5p in PDO#2, and could be rescued by GW4869 treatment in CAFs, or shFOXD3-AS1 in CAFs. (**L**) The results of RT-qPCR showing the effect of CAFs on the expression of miR-5586-5p in the orthotopic tumors of indicated group, and could be rescued by GW4869 treatment in CAFs, or shFOXD3-AS1 in CAFs. **P*<0.05, ***P*<0.01, ****P*<0.001.

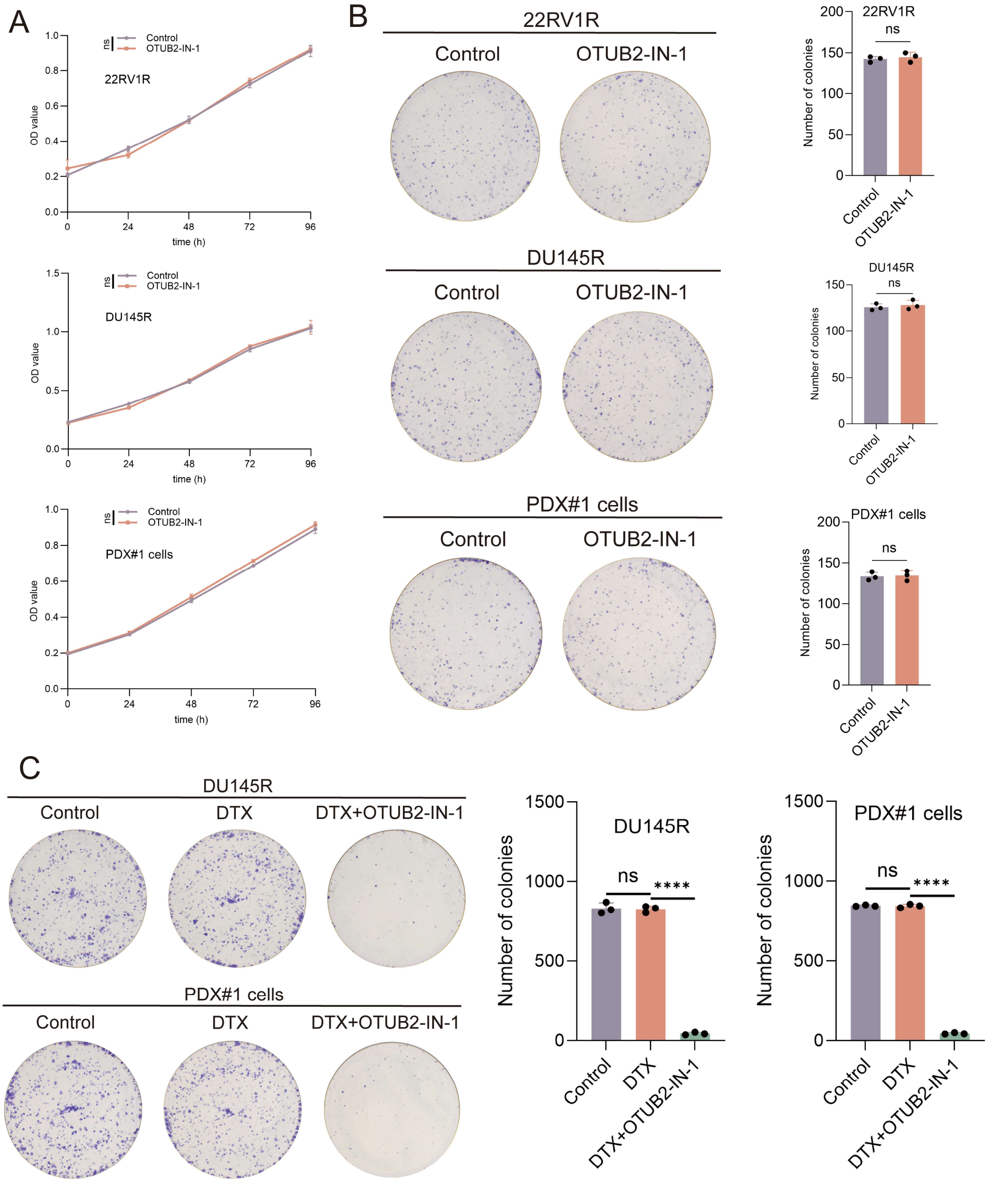

Figure S7. Validation of the non-specific toxicity of OTUB2-IN-1 and the effect of combination treatment with OTUB2-IN-1 and DTX.

(**A**) CCK8 assay showing the non-specific toxicity of OTUB2-IN-1 on the proliferation ability of DTX-resistant CRPC cells (22RV1, DU145, PDX#1 cells); three biological replicates were conducted; two-way repeated measures ANOVA test. (**B**) Representative images and quantitative results of cloning formation assay showing the non-specific toxicity of OTUB2-IN-1 on the cloning formation ability of DTX-resistant CRPC cells (22RV1, DU145, PDX#1 cells); three biological replicates were conducted; unpaired two-tailed student's t-test. (**C**) Representative images and quantitative results of cloning formation assay showing the effect of DTX combining with OTUB2-IN-1 on the cloning formation ability of DU145R and PDX#1 cells; three biological replicates were conducted; one-way ANOVA test. **P*<0.05, ***P*<0.01, ****P*<0.001.

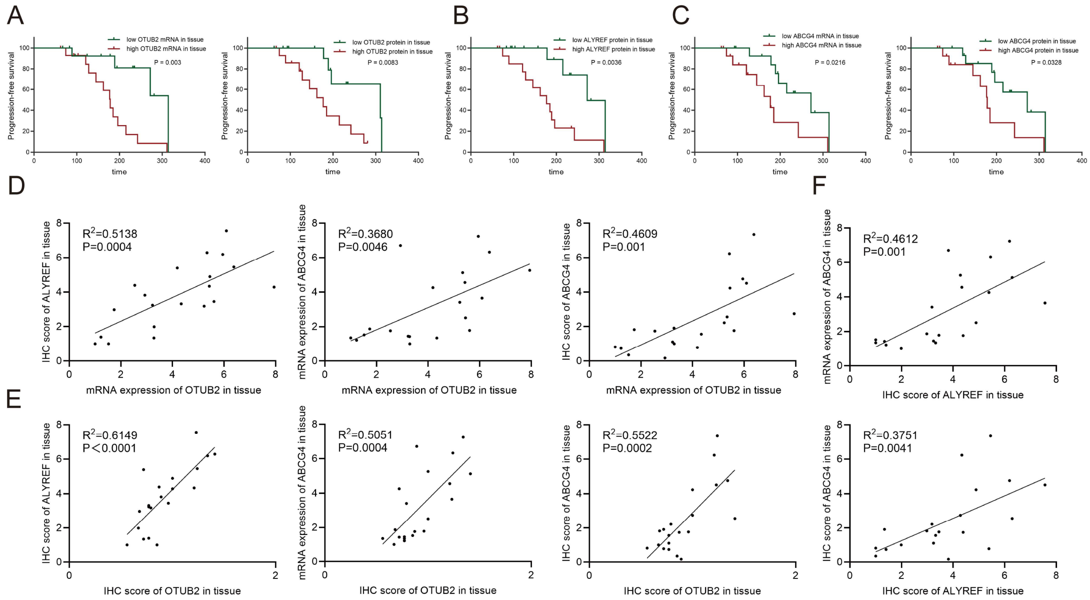

Figure S8. Validation of the OTUB2/ALYREF/ABCG4 axis on the DTX resistance using clinical samples.

(**A-C**) The results of Kaplan-Meier survival analysis showing the effect of OTUB2 mRNA, or OTUB2 protein, or ALYREF protein, or ABCG4 mRNA, or ABCG4 protein on the progression-free survival of CRPC patients receiving DTX chemotherapy. (**D**) The associations of OTUB2 mRNA expression with ALYREF protein (or ABCG4 mRNA, or ABCG4 protein) expression in the tissues of CRPC patients. (**E**) The associations of OTUB2 protein expression with ALYREF protein (or ABCG4 mRNA, or ABCG4 protein) expression in the tissues of CRPC patients. (**F**) The associations of ALYREF protein expression with ABCG4 mRNA (or ABCG4 protein) expression in the tissues of CRPC patients. **P*<0.05, ***P*<0.01, ****P*<0.001.

Table S1. Clinicopathological characteristics of 12 cases of prostate cancer tissue used for PDX construction.

ID	Pathological type	Age	Gleason score	PSA (ng/mL)	T stage	Castrate resistance	sample source	Metastasis

PDX#1	Adenocarcinoma	72	8	77.91	T4	Yes	prostate biopsy	bone, lymph node
PDX#2	Adenocarcinoma	78	9	59.72	T4	Yes	prostate biopsy	bone, lymph node
PDX#3	Adenocarcinoma	81	8	17.44	T3	No	radical prostatectomy	No
PDX#4	Adenocarcinoma	67	8	21.73	T3	No	radical prostatectomy	No
PDX#5	Adenocarcinoma	73	9	9.11	T3	No	radical prostatectomy	No
PDX#6	Adenocarcinoma	75	7	17.62	T2	No	radical prostatectomy	No
PDX#7	Adenocarcinoma	65	7	24.78	T4	No	radical prostatectomy	lymph node
PDX#8	Adenocarcinoma	61	9	14.91	T3	No	radical prostatectomy	No
PDX#9	Adenocarcinoma	69	8	18.29	T3	No	radical prostatectomy	No
PDX#10	Adenocarcinoma	77	8	36.27	T3	No	radical prostatectomy	No
PDX#11	Adenocarcinoma	79	10	48.31	T3	No	radical prostatectomy	No
PDX#12	Adenocarcinoma	71	9	55.73	T3	No	radical prostatectomy	No

Table S2. Clinicopathological characteristics of 30 cases of CRPC patients used for prognosis analysis

characteristics		Pathological type
Age		68(62-85)
Gleason		
	＜8	11
	⩾8	19
PSA		
	＜20	8
	⩾20	22
T stage		
	T3	12
	T4	18
Metastasis		
	lymph node	13
	bone	25
	visceral	7
Disease volume		
	low	9
	high	21
